# Skeletal muscle alterations and exercise performance decrease in erythropoietin-deficient mice: a comparative study

**DOI:** 10.1186/1755-8794-5-29

**Published:** 2012-06-29

**Authors:** Laurence Mille-Hamard, Veronique L Billat, Elodie Henry, Blandine Bonnamy, Florence Joly, Philippe Benech, Eric Barrey

**Affiliations:** 1 Unité de Biologie Intégrative des Adaptations à l’Exercice – INSERM 902, Genopole, F-91058, Evry, France; 2 GenoSciencePharma, 2, rue, Mascaron, F-13006, Marseille, France; 3 UMR1313 Génétique Animale et Biologie Intégrative, INRA, F-78350, Jouy-en-Josas, France

**Keywords:** Erythropoietin, Exercise, Skeletal muscle

## Abstract

**Background:**

Erythropoietin (EPO) is known to improve exercise performance by increasing oxygen blood transport and thus inducing a higher maximum oxygen uptake (VO_2max_). Furthermore, treatment with (or overexpression of) EPO induces protective effects in several tissues, including the myocardium. However, it is not known whether EPO exerts this protective effect when present at physiological levels. Given that EPO receptors have been identified in skeletal muscle, we hypothesized that EPO may have a direct, protective effect on this tissue. Thus, the objectives of the present study were to confirm a decrease in exercise performance and highlight muscle transcriptome alterations in a murine EPO functional knock-out model (the EPO-d mouse).

**Methods:**

We determined VO_2max_ peak velocity and critical speed in exhaustive runs in 17 mice (9 EPO-d animals and 8 inbred controls), using treadmill enclosed in a metabolic chamber. Mice were sacrificed 24h after a last exhaustive treadmill exercise at critical speed. The tibialis anterior and soleus muscles were removed and total RNA was extracted for microarray gene expression analysis.

**Results:**

The EPO-d mice’s hematocrit was about 50% lower than that of controls (p < 0.05) and their performance level was about 25% lower (p < 0.001). A total of 1583 genes exhibited significant changes in their expression levels. However, 68 genes were strongly up-regulated (normalized ratio > 1.4) and 115 were strongly down-regulated (normalized ratio < 0.80). The transcriptome data mining analysis showed that the exercise in the EPO-d mice induced muscle hypoxia, oxidative stress and proteolysis associated with energy pathway disruptions in glycolysis and mitochondrial oxidative phosphorylation.

**Conclusions:**

Our results showed that the lack of functional EPO induced a decrease in the aerobic exercise capacity. This decrease was correlated with the hematocrit and reflecting poor oxygen supply to the muscles. The observed alterations in the muscle transcriptome suggest that physiological concentrations of EPO exert both direct and indirect muscle-protecting effects during exercise. However, the signaling pathway involved in these protective effects remains to be described in detail.

## Background

The hematopoietic effect of erythropoietin (EPO) is known to increase exercise performance by inducing a higher maximal oxygen uptake (VO_2max_) in athletes. This EPO cytokine is also used as a therapeutic in patients. Indeed, disease-associated, low levels of EPO lead to a drop in hematocrit (Htc), marked anemia and decreased exercise performance. Congestive heart failure (CHF) and chronic kidney failure (CKF) lead to progressive renal dysfunction and a decrease in EPO levels, with reduced erythrocyte production by bone marrow. Thus, reduced exercise capacity is a clinical hallmark in CHF and CKF patients, who typically show lower VO_2max_ values than age-matched normal subjects. Indeed, as kidney function declines over time, there is a corresponding decline in exercise performance [1,2]. The normalization of hemoglobin concentration in these patients by administration of EPO-stimulating agents improves exercise capacity and raises VO_2max_ by increasing oxygen supply to muscles. Furthermore, the higher the increase in hemoglobin levels, the greater the improvement in exercise duration [3-6]. This effect continues at above-physiological levels of EPO. In cycling and running, the illegal administration of exogenous EPO is used to artificially increase performance levels (despite the occurrence of adverse effects). In addition, administration of recombinant human EPO (rHuEpo) was found to increase time to swimming exhaustion in a murine CKF model [7-9]. However, it is not known whether EPO directly affects muscle functions.

Furthermore, it is now well accepted that EPO’s biological activity is not restricted to regulation of erythropoiesis. In fact, EPO is a member of the cytokine superfamily and has significant homology with mediators of growth and inflammation. The EPO receptor (EPO-R) is also known to be present in heart, neuronal, retinal, renal and muscle tissues [10-13]. Unsurprisingly, additional biological functions for EPO have been identified. The synthetic form of EPO has been shown to exert remarkable, tissue-protective effects on several tissues. The broad efficiency of EPO depends on its key role in various protective pathways, such as inhibition of apoptosis, vascular restoration, attenuation of inflammatory responses and better overall function [10,14-18]. However, EPO’s protective role in muscle tissue has been mainly studied on cardiac muscle and *in vivo* and in various animal models [19]. It has been shown that (i) rHuEpo pre-treatment attenuates myocardial infarct size and (ii) EPO has a cardioprotective effect on ischemia-reperfusion injury in various species [20-25]. This effect was hematocrit-independent (*i.e.* directly related to EPO), since EPO improved cardiac function at a dose that did not increase the hematocrit [26].

However, it is not known whether physiological levels of EPO exert a protective role in skeletal muscles. In view of (i) the identification of EPO-R in muscle tissue and (ii) the known tissue-protective effects of EPO, we hypothesized that physiological levels of the cytokine may have protective effects on muscle.

Thus, we used a murine EPO functional knock-out model (the EPO-d mouse, based on EPO immunization) to decrease active circulating levels of EPO and thus investigate the loss of function's impact on exercise performance and on the muscle transcriptome. The EPO-d mice had a low Htc and so we expected them to have lower performance levels and more impaired muscle oxidative function than inbred, control mice. Hence, the objectives of the present study were to confirm a decrease in exercise performance and highlight muscle transcriptome alterations in a murine EPO functional knock-out model (the EPO-d mouse).

## Methods

### Ethical approval

All protocols were approved by our institution’s Animal Care and Use Committee and complied with the Council of Europe’s European Convention for the Protection of Vertebrate Animals Used for Experimental and Other Scientific Purposes.

The protein functional knock-out was obtained by immunoneutralization of circulating EPO, according to the vaccination method developed by Nokad® [27]. Briefly, when immunization is performed with a modified self-protein like EPO, cross-reactive neutralizing antibodies are secreted and deplete the circulating protein. Repeated injections of the modified protein modulate the immune response and, in the present case, enabled us to study the effects of the loss of active, circulating EPO and the subsequent drop in Htc (US patent 2008/0220015A1). We always checked each EPO-d mouse’s Htc (down to as low as 20%) before initiating the exercise tests.

A total of 17 adult female C57Bl6/J mice (9 EPO-d mice and 8 control inbred animals) were included in this study. They were ear–punched for identification. Male were excluded to avoid a potential gender effect.

The mice were five months old when they performed the exercise tests. The animals were kept in an animal facility (CERFE, Genopole, Evry, France) in a specific and opportunist pathogen-free environment and at a temperature of 22°C with 12h:12h light-dark cycles. The animals were supplied with water and food *ad libitum*.

### Exercise protocols

#### Devices

The exercise testing protocol was performed on a single-lane motorized treadmill (Columbus Instruments, Columbus, OH) with an adjustable belt speed (0–99.9 m.min^-1^). The rear of the treadmill was equipped with a low-voltage, electric stimulating bar, to encourage each mouse to run. The bar was set to deliver 0.2 mA at a frequency of 0.25 Hz, which caused an uncomfortable shock but did not injure the animal.

#### Measurements and data recording

Oxygen consumption (VO_2_) was measured by means of a rapid-flow, open-circuit, indirect calorimeter. The single-lane test treadmill was placed in a metabolic chamber. Ambient air was fed through the chamber at a rate of 0.66 l.min^-1^; the flow was chosen such that the O_2_ difference across the chamber was within the sensor’s range (-0.3 to -0.8% O_2_). A fan mixed the incoming air with the air around the treadmill and blew it towards the animal. The air flowed from the front of the treadmill to the rear and then returned under the belt towards the front. This created a rapid, circular "loop" of mixed gases (incoming "fresh" air and accumulated exhaled gases) from which a sample was drawn for analysis (the Oxymax from Columbus Instruments). Gas samples were taken every 5s and dried prior to measurement of the oxygen and carbon dioxide fractions.

The gas analyzers were calibrated with standardized gas mixtures (Air Products, Paris, France) before every test session, as recommended by the manufacturer. The treadmill test provided an estimate of VO_2max_, defined as the highest oxygen consumption attained over a 15-second period during the testing protocol. To allow rapid comparison over a wide range of body weights (and especially with human data), dimensional analysis and empirical studies show that VO_2_ should be expressed in relation to body mass raised to the power of 0.75. [28,29].

#### Familiarization

The mice were familiarized with the treadmill over a one-week period via the completion of four 10-min running sessions from 0 to 9 m.min^-1^ (0, 3, 6 and 9 m.min^-1^). All mice succeeded in running for the required time at an intensity of 9 m.min^-1^. The velocity was not increased above this value, in order to avoid a training effect. The mice subsequently performed an incremental exercise test.

#### Incremental test load: Peak velocity, blood lactate and $\dot{\text{V}}{\text{O}}_{2}$_max_ determination

Starting from a speed of 10 m.min^-1^, the exercise intensity was increased by 3 m.min^-1^ every 3 min, with an incline of 0%. Exercise continued until exhaustion, which was defined as an inability to maintain the running speed despite contact with the electric grid for more than 5 sec. All measurements were made by the same investigator. The last stage completed by the mouse was defined as the peak velocity (vPeak). Blood lactate levels were measured before the exercise and two minutes after the end of the exercise ([La]_INC_). After local antisepsis with alcohol, the distal 2mm of the animal’s tail was cut off. To avoid contamination, the first drop of blood was discarded. Next, a 5 μl blood sample was collected on a small test strip and the blood lactate concentration was measured with a Lactate Pro assay (Arkray Inc., Kyoto, Japan).

#### Steady-state intensity exercise: The critical speed determination

The protocol consisted of four constant-speed runs (18–51 m/min) leading to exhaustion within one hour, as described in an earlier study [30]. A single trial was performed per day, and the protocol covered a period of 4 days. The time to exhaustion was recorded at each speed. Two parameters were used to estimate endurance performance: the distance the mice were able to cover at a given speed (limit distance, in meters) and the time needed to cover the distance (limit time, seconds). The critical speed (CS) was calculated, from the slope (*a*) of the regression line, plotting the distance (y) vs. the time to exhaustion (x) from the four tests, according to the equation *y* = *ax* + *b*.

#### Exhaustive exercise at the critical speed and muscle collection

At least two days after the end of the determination of CS, vPeak and VO_2max_, all animals ran at their individual CS until exhaustion. We recorded the time to exhaustion and the blood lactate concentrations before and 2 min after the end of exercise ([La]_SS_). Mice were sacrificed by cervical dislocation 24h later. The tibialis anterior and soleus muscles were rapidly removed, immerged for 2 hours in RNAlater reagent (Ambion, Austin, TX) and then frozen in liquid nitrogen. The samples were then stored at -80°C prior to RNA extraction for microarray and RT-qPCR analysis.

### Muscle gene expression analysis

#### Production of the murine long oligonucleotide microarray

Gene expression analysis was performed using a 25K mouse long oligonucleotide microarray [Gene Expression Omnibus accession number GPL8349: http://www.ncbi.nlm.nih.gov/geo/query/acc.cgi?acc=GPL8349, [31]]. The microarrays were spotted (LEFG, CEA, Evry, France) by printing the probes (suspended in a spotting buffer composed of 1:1 v/v dimethyl sulfoxide (DMSO) and Tris EDTA buffer) on hydrogel slides (Nexterion, Schott) with a Microgrid-II robot (Genomic Solutions Inc., Ann Arbor, MI).

#### RNA extraction

Total RNA was extracted from each muscle sample using a phenol-chloroform method (TRIzol® reagent, Invitrogen Life Technologies, Carlsbad, CA, USA). The quality of total RNA was verified by micro channel electrophoresis (RNA 6000 Nano LabChip, Bioanalyzer®, Agilent, Agilent technologies, Santa Clara, USA). The total RNA concentration and purity were measured by optical density with a spectrophotometer according to the absorbance at 260 nm and the 260nm/280nm absorbance ratio, respectively (Nanodrop®, Thermo Scientific, Wilmington, DE, USA). RNA was considered as pure when the 260nm/280nm absorbance ratio was close to 2. The total RNA extracts were stored at -80°C prior to microarray analysis.

### Microarray hybridization and signal quantification

#### Microarray hybridization

The tibialis anterior RNA extracts from each mouse were pooled into the corresponding EPO-d and control groups. Likewise, soleus muscle RNA extracts were pooled into an EPO-d group and a control group. Eight mouse 25K microarrays were used to analyze the samples using a dye-swap design.(2 groups x 2 muscles x 2). Briefly, the hybridization protocol [32] included the following steps: after purification of the amino-modified RNA, monofunctional forms of cyanine-3 (Cy3) or cyanine-5 (Cy5) fluorochrome were coupled to 5 μg each of the test and control samples using an indirect method: the fluorochromes’ ester groups were bound to aminoallyl groups incorporated into the RNA during *in vitro* transcription. The synthesis of RNA with a polyA tail was performed using *in vitro* transcription with aminoallyl UTP. A total RNA (1 μg) was used to start the synthesis using the *in vitro* transcription kit according to the manufacturer’s protocol (Aminoallyl MessageAmp ® II aRNA, Invitrogen Life Technologies, Carlsbad, CA, USA). This method equally increased the number of mRNA copies of each transcript without changing the proportion of the initial population of transcripts. The purity and concentration of aminoallyl RNA (aaRNA) were again checked by microchannel electrophoresis (with a RNA 6000 Nano LabChip in a Bioanalyzer) and spectrophotometry (Nanodrop). The aaRNAs were stored at -80°C. The marked targets were then mixed and hybridized on the same chip for immediate comparison of the two nucleic acid populations. For each sample, a dye-swap (duplicate measurement) was performed to avoid asymmetry of marker affinity (Cy3 and Cy5). The fluorescence intensities of Cy3 (532 nm) and Cy5 (635 nm) were imaged separately with a laser scanner (GenePix 4000B, Molecular Device, Sunnyvale, CA, USA). The resulting tiff images (10 μm/pixel) were analyzed using dedicated microarray image analysis software (GenePix Pro 6.0 software, Molecular Device, Sunnyvale, CA, USA) that located each spot automatically (with manual control) and quantified its fluorescence intensity. For each microarray, the data output were the fluorescence intensities of all the spots corresponding to each gene. These data were imported into genomic data analysis software (Gene Spring software, Agilent Technologies, Santa Clara, CA, USA) to normalize the data before filtering and statistical analysis. For each sample, a duplicate analysis (dye-swap) was made and the average fluorescence results were used. Next, all the results were normalized per spot and per microarray with a locally weighted scatterplot smoothing regression method, in order to remove systematic variations. Lastly, the expression ratio was obtained for each muscle and each gene.

Ratio = fluorescence in EPO-d muscle / fluorescence in the corresponding control muscle

For statistical analysis, a log transformation ratio was used to normalize the distribution. A list of the significant genes were obtained by filtering the genes with a cut-off T-test (p-value < 0.05). Benjamini and Hochberg multiple testing correction was used to avoid a biased discovery rate [33] The Student t-test detected the mean expression ratio of replicates that differed significantly from one (i.e. an expression ratio of 1 means that there was no change in gene expression).

### Real-time, quantitative PCR (RT-qPCR)

In order to validate the most significant results from the microarray analysis, quantitative RT-qPCRs were conducted in triplicate for the top ten up- and down-regulated gene transcripts in muscles from both EPO -d and control inbred mice. The primers were designed using Primer express software (Applied Biosystems, Life Technologies, Carlsbad, CA, USA) (Table 1). All reactions were carried out in a 7500 Real-Time PCR System (Applied Biosystems). Briefly, reverse transcription was performed on 20 ng of the aaRNA used for microarray hybridization. Random primers (100 ng) and 10 pmol of dATP, dCTP, dGTP and dTTP were added to RNA in a 13 μL final volume. The reaction tubes were incubated at 70°C for 5 min. The mixture was incubated with random annealing primer at room temperature (~25°C) for 10 min. Four microliters of 5x first-strand buffer, 200 pmol of DTT and 200U of Superscript II reverse transcriptase were added to the tube to give a 20μL final volume and incubated at 42°C for 1 hour. The enzyme was inactivated by incubating the mixture at 70°C for 15 min. The cDNA concentration was measured in terms of the optical density at 260 and 280 nm (Nanodrop ®). The cDNA solution was diluted in water to obtain a final concentration of 20 ng/μL and stored at -20°C. Complementary DNA (100 ng) obtained from aaRNA was amplified in a 20 μL PCR reaction with 10 μmol of each specific primer and 10μL SYBR® Green PCR Master Mix (Qiagen, Hilden, Germany). Amplification steps were as follows: denaturation for 10 min at 95°C, 40 cycles of denaturation at 95°C for 15 s and annealing extension at 60°C for 1 min. The comparative threshold cycle (Ct) method was used to calculate the amplification, as specified by the manufacturer. The expression results of the RT-qPCR for muscles from EPO-d and control inbred mice were normalized against the mean Ct for the endogenous gene Cish2 (measured in all muscle samples and found to independent of the EPO deficiency). Each gene’s relative expression ratio in the EPO-d mice was calculated according to the expression of the corresponding gene in the control mice.

**Table 1:** List of the primers used for RT-qPCR

**Genes**	**Primers**	**Sequences 5'- > 3'**	**Length**	**Tm**	**%GC**
Mcptl	Fwd	TTCTGGACGCGGAAATGC	18	59	56
	Rev	CTGTTAATCCAGGGCACATGTG	22	59	50
Runx1	Fwd	GCCATTTTGGGTCTGCTTTC	20	58	50
	Rev	TTGTCTGCGCAGGACGTTT	19	59	53
Plcb2	Fwd	ACCGCATTGATGTGGTGGTA	20	58	50
	Rev	TGCGTACACTGCGCTCTGA	19	59	58
Ccdc130	Fwd	GGTGAAAGGAAAGGCCAGAAC	21	59	52
	Rev	ATCGGTTGAGAGAGCCATGCT	21	60	52
Chfr	Fwd	AAGGAAGCCATGCCATATGC	20	59	50
	Rev	GGATCCTGCTCCCGTTCA	18	58	61
Ints1	Fwd	GGACTGCCTGCGATCCTTTA	20	59	55
	Rev	GCCCACGTACTTGTGGATGA	20	58	55
Pip4k2b	Fwd	CCGATTCTCAGCGTCCTGAT	20	59	55
	Rev	CATCCGGCATTAGCATGACA	20	59	50
Isg20l2	Fwd	CCAGGGAGACACATGCAGTAGAC	23	60	57
	Rev	CCTCTCCCCAGTGATTCTAGCTT	23	59	52
Ckm	Fwd	GCTGACCCCTGACCTCTACAATA	23	58	52
	Rev	CACCCCAGTCTGGATGACATC	21	59	57
Eno3	Fwd	GCAATTGCCTGCTCCTGAAG	20	60	55
	Rev	GTGCAAGTTTACAGGCCTGGAT	22	59	50
Serpinb2	Fwd	CAGGCACAAGCAGGAGATAAAA	22	58	45
	Rev	CCCCTGTGGTGTGTTGATTG	20	58	55
Hspb7	Fwd	AGCTGATGGCACAGTCATGAAC	22	59	50
	Rev	GCCGAGGTCACCGATGTT	18	58	61
Chac1	Fwd	GGTCATTGCCACACAGATCCT	21	58	52
	Rev	CTGCCAAACGCAATAAGTACTCA	23	58	43
Nrap	Fwd	GCTTTCTGTTAATAACTTCGTGAGTCA	27	58	37
	Rev	GACGCTAGTGAACGTGTTGTTCTT	24	59	46
Zp2	Fwd	TGACTGCTGGGCAACTTCTTC	21	59	52
	Rev	TCACAGCCATCCATGACAATCT	22	59	45
Atp1a2	Fwd	CATAAGGCTCAGGAGTCACAGAAG	24	58	50
	Rev	CACAGGGAGAAGTGGCAGCTA	21	59	57
Ctsd	Fwd	GGCGTCTTGCTGCTCATTCT	20	59	55
	Rev	ACTTGCGCAGAGGGATTCTG	20	59	55
Socs3	Fwd	GCCACCTGGACTCCTATGAGAA	22	59	55
	Rev	GGAGCATCATACTGATCCAGGAA	23	59	48
Cish2 (endogenous	Fwd	TCCAGATGTGCAAGGATAAACG	22	59	45
gene)	Rev	GGTTTGGTCAGGTACAGGTGAAC	23	58	52
Erk1	Fwd	CCTGCTGGACCGGATGTTA	19	58	58
	Rev	TGAGCCAGCGCTTCCTCTA	19	58	58
Epor	Fwd	TTGGTGTGTTTCTGGGAGGAA	21	59	48
	Rev	ACCCTCGAGCTGGTATGAGAAG	22	58	55
Hif1a	Fwd	TGCATCTCCATCTTCTACCCAAGT	24	60	46
	Rev	CCGACTGTGAGTGCCACTGTAT	22	59	55
Vegf	Fwd	GCAGGCTGCTGTAACGATGA	20	59	55
	Rev	CATGATCTGCATGGTGATGTTG	22	58	45

### Data mining

A biological interpretation of the data was performed using two data-mining programs to identify the main biological functions and the main metabolic pathway disorders revealed by the expression profile in EPO-d muscles. Ingenuity® Pathway Analysis ( http://www.ingenuity.com) was used to identified the biological functions that were the most significantly associated with the gene list results. Significant genes whose functions had been described in published papers were considered for the analysis. Fischer’s exact test was used to calculate a p-value for the probability with which each biological function assigned to a data set was due to chance alone.

The analysis of the main metabolic pathways and their potential dysfunctions was performed using Predictsearch® software written in Java/Perl (Prediguard, http://prediguard.com/predictsearch.htm), which has been previously described in [34]. This software characterizes the pathways and functional networks in which the selected genes are involved. Briefly, gene aliases corresponding to the significant modulated genes were submitted as queries to PubMed in order to collect titles and abstracts of all related publications. The results are presented in a graph that shows the relationships between each gene and each cell type, cellular compartment and biological function.

### Statistics

A Mann-Whitney U test was applied to compare variables in EPO-d mice with those in control mice variables. A Spearman test was used to test correlations between exercise parameters. A three-way analysis of variance was used to test the effects of EPO deficiency, muscle and gene on the RT-qPCR Ct values.

## Results

### Exercise physiology

The two groups of mice did not differ significantly in terms of age and weight at baseline. The EPO-d group showed a two-fold decrease in Htc, relative to the control group (19.56 ± 2.74% and 40.75 ± 2.71%, respectively; p < 0.0001). The VO_2max_, vPeak, and CS performance indexes were lower in the EPO-d group than in the control group. However, the two groups did not differ in terms of the time to exhaustion at the CS (Tlim). The [La]_INC_ measured at the end of the incremental exercise was higher in the EPO-d group, whereas the [La]_SS_ values measured at the end of steady-state exercise at the CS were similar (Table 2).

**Table 2:** Physiologic variables and metabolic indexes during exercise in control and EPO-deficient mice

	**Control (n = 8)**	**EPO-d (n = 9)**	**p-value**
**Age (weeks)**	21.3±3.7	19.3±1.0	NS
**Weight (g)**	22.7±0.9	21.7±1.28	NS
**Htc (%)**	40.8±2.7	19.6±2.7	0.001
**vPeak (m/min)**	19.0±3.2	13.9±2.3	0.004
**CS (m.min**^**-1**^**)**	16.4±2.9	11.15±1.5	0.002
**VO**_**2max**_**(ml.kg**^**-0.75**^**.min**^**-1**^**)**	48.3±1.4	34.4±5.4	0.0005
**[La]**_**INC**_**(mmol.l**^**-1**^**)**	3.3±1.1	9.4±2.6	0.007
**Tlim (min)**	43.2±13.3	50.3±20.8	NS
**[La]**_**SS**_**(mmol.l**^**-1**^**)**	2.8±2.0	3.4±2.7	NS

Data are quoted as the mean ± SD; NS: not significant at p < 0.05; $\dot{\text{V}}{\text{O}}_{2}$: oxygen consumption; $\dot{\text{V}}{\text{O}}_{2}$_max_ :maximum $\dot{\text{V}}{\text{O}}_{2}$.

**Figure 1:**
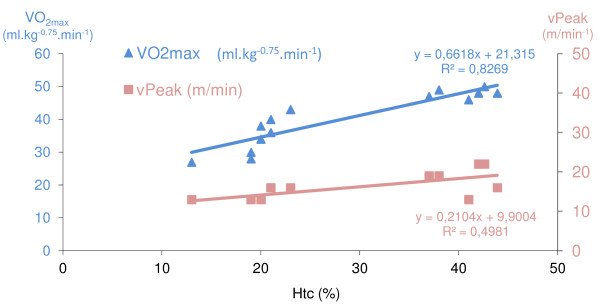
**Correlations between hematocrit on one hand and peak velocity and VO**_**2max **_**on the other**.

The VO_2max_ and vPeak were correlated with the Htc in each group separately and when pooled (p < 0.05) (Figure 1). In the control group, there was a correlation between the Htc and the CS (r =0.828 , p < 0.05).

### Gene expression in skeletal muscles

Thirty-three percent of the microarray probes (especially those for mitochondrial genes) hybridized with a significantly high fluorescence value. All the data from the present study have been deposited in the Gene Expression Omnibus (accession number pending). After filtering the normalized expression ratios with a cut-off at p < 0.05 (i.e. a significant difference from 1), a total of 1583 genes exhibited significant changes of expression levels between the EPO-d and the control mice ( Additional file 1: Table S1). Most of the changes were moderate (0.80 < normalized ratio < 1.4). However, 68 genes were strongly up-regulated genes (normalized ratio > 1.4) and 115 were strongly down-regulated (normalized ratio <0.80) (Tables 3 and 4).

**Table 3:** List of significantly up-regulated genes expressed in the tibialis anterior and soleus muscles in EPO-deficient mice and control mice

**Genes**	**Entrez Gene ID**	**Normalized ratio**	**t-test P-value**	**Description**
Mcptl	17233	3.25	0.01	mast cell protease-like
Dhrs4	28200	2.55	0.01	dehydrogenase/reductase (SDR family) member 4
Runx1	12394	1.88	0.03	runt related transcription factor 1
3830408D24Rik	100039781	1.87	0.01	RIKEN cDNA 3830408D24 gene
Plcb2	18796	1.87	0.04	phospholipase C, beta 2
1810013L24Rik	69053	1.79	0.05	RIKEN cDNA 1810013L24 gene
Aldh8a1	237320	1.78	0.02	aldehyde dehydrogenase 8 family, member A1
Asp-ARNt_TRND	4555	1.76	0.01	mitochondrially encoded tRNA aspartic acid [ Homo sapiens ]
Ccdc130	67736	1.74	0.02	coiled-coil domain containing 130
Chfr	231600	1.71	0.02	checkpoint with forkhead and ring finger domains
Ints1	68510	1.71	0.02	integrator complex subunit 1
Pip5k2b	108083	1.70	0.02	phosphatidylinositol-5-phosphate 4-kinase, type II, beta
Isg20l2	229504	1.70	0.01	interferon stimulated exonuclease gene 20-like 2
Bace1	23821	1.69	0.04	beta-site APP cleaving enzyme 1
1200015F23Rik	67809	1.68	0.02	RIKEN cDNA 1200015F23 gene
Gmfg	63986	1.67	0.02	glia maturation factor, gamma
BC037032	414066	1.65	0.01	cDNA Sequence BC037032
Zdhhc9	208884	1.65	0.03	zinc finger, DHHC domain containing 9
Metap2	56307	1.62	0.02	methionine aminopeptidase 2
1110008L16Rik	66132	1.61	0.03	RIKEN cDNA 1110008L16 gene
Asn-ARNt_TRNN	4570	1.61	0.00	mitochondrially encoded tRNA asparagine [ Homo sapiens ]
Ube2g1	67128	1.61	0.01	ubiquitin-conjugating enzyme E2G 1 (UBC7 homolog, C. elegans)
4933409D19Rik	71084	1.61	0.05	RIKEN cDNA 4933409D19 gene
Tyr-ARNt_TRNY	4579	1.60	0.03	mitochondrially encoded tRNA tyrosine
Mif	17319	1.60	0.04	macrophage migration inhibitory factor
Olfr1428	258673	1.60	0.03	olfactory receptor 1428
Cygb	114886	1.60	0.03	cytoglobin
Olfr1387	258465	1.59	0.01	olfactory receptor 1387
Adarb1	110532	1.58	0.01	adenosine deaminase, RNA-specific, B1
BC023105	207269	1.58	0.02	cDNA sequence BC023105
Aak1	269774	1.56	0.02	AP2 associated kinase 1
9830134C10Rik	442827	1.56	0.00	RIKEN cDNA 9830134C10 gene
4921530L21Rik	66732	1.55	0.04	RIKEN cDNA 4921530L21 gene
A930014E01Rik	78598	1.52	0.03	RIKEN cDNA A930014E01 gene
Dgkh	380921	1.51	0.04	diacylglycerol kinase, eta
Ccdc72	66167	1.51	0.05	coiled-coil domain containing 72
V1rc1	113858	1.51	0.00	vomeronasal 1 receptor, C1
Zim1	22776	1.51	0.01	zinc finger, imprinted 1
Ace	11421	1.50	0.04	angiotensin I converting enzyme (peptidyl-dipeptidase A) 1
Xrcc6	14375	1.50	0.01	X-ray repair complementing defective repair in Chinese hamster cells 6
Dcun1d3	233805	1.50	0.02	DCN1, defective in cullin neddylation 1, domain containing 3 (S. cerevisiae)
Adam28	13522	1.49	0.04	a disintegrin and metallopeptidase domain 28
Adamts10	224697	1.49	0.03	a disintegrin-like and metallopeptidase (reprolysin type) with thrombospondin type 1 motif, 10
Bach2	12014	1.46	0.03	BTB and CNC homology 2
Cbl	12402	1.46	0.01	Casitas B-lineage lymphoma
Hist1h2an	319170	1.46	0.02	histone cluster 1, H2an
Eif5a	276770	1.45	0.05	eukaryotic translation initiation factor 5A
Zcchc2	227449	1.45	0.02	zinc finger, CCHC domain containing 2
Stk24	223255	1.45	0.05	serine/threonine kinase 24 (STE20 homolog, yeast)
B4galt3	57370	1.45	0.02	UDP-Gal:betaGlcNAc beta 1,4-galactosyltransferase, polypeptide 3
Tprkb	69786	1.44	0.00	Tp53rk binding protein
Sfpq	71514	1.44	0.05	splicing factor proline/glutamine rich (polypyrimidine tract binding protein associated)
Foxo1	56458	1.44	0.02	forkhead box O1
4933436I01Rik	66780	1.44	0.03	RIKEN cDNA 4933436I01 gene
9830107B12Rik	328829	1.43	0.01	RIKEN cDNA 9830107B12 gene
Dhx30	72831	1.43	0.02	DEAH (Asp-Glu-Ala-His) box polypeptide 30
Sema3e	20349	1.43	0.01	sema domain, immunoglobulin domain (Ig), short basic domain, secreted, (semaphorin) 3E
Vps37b	330192	1.42	0.03	vacuolar protein sorting 37B (yeast)
AI662250	106639	1.41	0.05	expressed sequence AI662250
Arhgap26	71302	1.41	0.01	Rho GTPase activating protein 26
4930434E21Rik	381693	1.41	0.04	RIKEN cDNA 4930434E21 gene
1500001A10Rik	68955	1.41	0.02	RIKEN cDNA 1500001A10 gene
Prtg	235472	1.40	0.00	protogenin homolog (Gallus gallus)
Lefty2	320202	1.40	0.02	Left-right determination factor 2
4933431J24Rik	71298	1.40	0.00	RIKEN cDNA 4933431J24 gene
Opn1sw	12057	1.40	0.01	opsin 1 (cone pigments), short-wave-sensitive (color blindness, tritan)
Vcpip1	70675	1.40	0.01	valosin containing protein (p97)/p47 complex interacting protein 1
Defb37	353320	1.40	0.01	defensin beta 37

A p-value <0.05 indicates that the expression ratio differed significantly from 1.

**Table 4:** List of significantly down-regulated genes expressed in the tibialis anterior and soleus muscles in EPO-d mice and control mice

**Genes**	**Entrez Gene ID**	**Normalized ratio**	**t-test P-value**	**Description**
Ckm	12715	0.52	0.00	creatine kinase, muscle
Eno3	13808	0.53	0.01	enolase 3, beta muscle
Serpinb2	18788	0.57	0.04	serine (or cysteine) peptidase inhibitor, clade B, member 2
Apool	68117	0.58	0.02	apolipoprotein O-like
Hspb7	29818	0.58	0.03	heat shock protein family, member 7 (cardiovascular)
Chac1	69065	0.60	0.04	ChaC, cation transport regulator-like 1 (E. coli)
Cln3	12752	0.61	0.01	ceroid lipofuscinosis, neuronal 3, juvenile (Batten, Spielmeyer-Vogt disease)
Nrap	18175	0.63	0.00	nebulin-related anchoring protein
Zyg11b	414872	0.63	0.00	zyg-ll homolog B (C. elegans)
Zp2	22787	0.65	0.00	zona pellucida glycoprotein 2
Atp1a2	98660	0.66	0.01	ATPase, Na+/K + transporting, alpha 2 polypeptide
Ctsd	13033	0.66	0.00	cathepsin D
Lrrc42	77809	0.66	0.01	leucine rich repeat containing 42
Ppp3ca	19055	0.67	0.01	protein phosphatase 3, catalytic subunit, alpha isoform
1600014E20Rik	71995	0.67	0.04	endogenous retroviral sequence 3
Kcnb1	16500	0.67	0.02	potassium voltage gated channel, Shab-related subfamily, member 1
1700052M18Rik	78415	0.68	0.04	RIKEN cDNA 1700052M18 gene
C1qtnf4	67445	0.68	0.03	C1q and tumor necrosis factor related protein 4
Setd3	52690	0.68	0.05	SET domain containing 3
Rtn2	20167	0.69	0.01	reticulon 2 (Z-band associated protein)
1110018H23Rik	68509	0.69	0.01	RIKEN cDNA 1110018H23 gene
Mgat2	217664	0.69	0.03	mannoside acetylglucosaminyltransferase 2
Smpd1	20597	0.70	0.02	sphingomyelin phosphodiesterase 1, acid lysosomal
Tbcc	72726	0.71	0.02	tubulin-specific chaperone c
Zfand5	22682	0.72	0.01	zinc finger, AN1-type domain 5
Tuba8	53857	0.72	0.02	tubulin, alpha 8
Fndc5	384061	0.73	0.02	fibronectin type III domain containing 5
Magea5	17141	0.73	0.04	melanoma antigen, family A, 5
Actg2	11468	0.73	0.03	actin, gamma 2, smooth muscle, enteric
Galnt14	71685	0.74	0.01	UDP-N-acetyl-alpha-D-galactosamine:polypeptide N-acetylgalactosaminyltransferase 14
2610019E17Rik	75614	0.74	0.01	RIKEN cDNA 2610019E17 gene
Scn4b	399548	0.74	0.00	sodium channel, type IV, beta
Olfr1370	258528	0.74	0.03	olfactory receptor 1370
Cbln2	12405	0.74	0.02	cerebellin 2 precursor protein
4933406C10Rik	74076	0.74	0.04	RIKEN cDNA 4933406C10 gene
Zfp618	72701	0.75	0.00	zinc fingerprotein 618
Slc2a4	6517	0.75	0.01	solute carrier family 2 (facilitated glucose transporter), member 4 [ Homo sapiens ]
Stx3	20908	0.75	0.01	syntaxin 3
Mdh2	4191	0.75	0.03	malate dehydrogenase 2, NAD (mitochondrial) [ Homo sapiens ]
Chchd6	66098	0.75	0.05	coiled-coil-helix-coiled-coil-helix domain containing 6
Gpsn2	106529	0.75	0.00	glycoprotein, synaptic 2
D230014K01Rik	217364	0.76	0.02	RIKEN cDNA D230014K01 gene
Zfp286	192651	0.76	0.04	zinc finger protein 286
Pctk1	18555	0.76	0.03	PCTAIRE-motif protein kinase 1
1700008N11Rik	75442	0.76	0.03	RIKEN cDNA 1700008N11 gene
1700015C15Rik	75532	0.76	0.02	RIKEN cDNA 1700015C15 gene
Uqcrc1	22273	0.76	0.00	ubiquinol-cytochrome c reductase core protein 1
Abi3	66610	0.77	0.02	ABI gene family, member 3
Vat1	26949	0.77	0.00	vesicle amine transport protein 1 homolog (T californica)
Snw1	66354	0.77	0.04	SNW domain containing 1
Eef1a2	13628	0.77	0.01	eukaryotic translation elongation factor 1 alpha 2
Nme5	75533	0.77	0.01	expressed in non-metastatic cells 5
Fxyd1	56188	0.77	0.03	FXYD domain-containing ion transport regulator 1
1700039E22Rik	73322	0.77	0.01	RIKEN cDNA 1700039E22 gene
Herpud1	64209	0.77	0.02	homocysteine-inducible, endoplasmic reticulum stress-inducible, ubiquitin-like domain member 1
Pms2	18861	0.77	0.04	postmeiotic segregation increased 2 (S. cerevisiae)
Gli3	14634	0.77	0.05	GLI-Kruppel family member GLI3
Eef2	13629	0.78	0.01	eukaryotic translation elongation factor 2
Perld1	320655	0.78	0.03	per1-like domain containing 1
Pbxip1	229534	0.78	0.01	pre-B-cell leukemia transcription factor interacting protein 1
Ncf1	17969	0.78	0.03	neutrophil cytosolic factor 1
Olfr745	258296	0.78	0.03	olfactory receptor 745
Wfikkn2	278507	0.78	0.01	WAP, follistatin/kazal, immunoglobulin, kunitz and netrin domain containing 2
Syngr2	20973	0.78	0.01	synaptogyrin 2
Eml4	78798	0.78	0.04	echinoderm microtubule associated protein like 4
4930564G21Rik	75321	0.78	0.03	RIKEN cDNA 4930564G21 gene
B230217C12Rik	68127	0.78	0.02	RIKEN cDNA B230217C12 gene
1810054G18Rik	75660	0.78	0.00	lin-37 homolog (C. elegans)
Psmd4	19185	0.78	0.01	proteasome (prosome, macropain) 26S subunit, non-ATPase, 4
Rab14	68365	0.78	0.04	RAB14, member RAS oncogene family
Slc15a4	100561	0.78	0.03	solute carrier family 15, member 4
Rcl1	59028	0.79	0.03	RNA terminal phosphate cyclase-like 1
Ndufa3	66091	0.79	0.01	NADH dehydrogenase (ubiquinone) 1 alpha subcomplex, 3
Tpmt	22017	0.79	0.02	thiopurine methyltransferase
Twf2	23999	0.79	0.01	twinfilin, actin-binding protein, homolog 2 (Drosophila)
Rbpjl	19668	0.79	0.01	recombination signal binding protein for immunoglobulin kappa J region-like
Gngt2	14710	0.79	0.04	guanine nucleotide binding protein (G protein), gamma transducing activity polypeptide 2
Lmln	239833	0.79	0.03	leishmanolysin-like (metallopeptidase M8 family)
Olfr1433	258680	0.79	0.01	olfactory receptor 1433
2210404E10Rik	72305	0.79	0.02	RIKEN cDNA 2210404E10 gene
Sod1	20655	0.79	0.05	superoxide dismutase 1, soluble
2310007L24Rik	75573	0.79	0.02	RIKEN cDNA 2310007L24 gene
Asrgl1	66514	0.79	0.00	asparaginase like 1
Wdr54	75659	0.79	0.02	WD repeat domain 54
Ociad2	433904	0.79	0.01	OCIA domain containing 2
Atg9a	245860	0.79	0.05	autophagy-related 9A (yeast)
Rbm38	56190	0.79	0.01	RNA binding motif protein 38
Psmb5	19173	0.79	0.02	proteasome (prosome, macropain) subunit, beta type 5
Slc30a9	109108	0.79	0.03	solute carrier family 30 (zinc transporter), member 9
Agr2	23795	0.79	0.02	anterior gradient 2 (Xenopus laevis)
Cpne3	70568	0.79	0.01	copine III
Tbc1d1	57915	0.80	0.03	TBC1 domain family, member 1
Cacng6	54378	0.80	0.04	calcium channel, voltage-dependent, gamma subunit 6
5730494M16Rik	66648	0.80	0.01	RIKEN cDNA 5730494M16 gene
Olfr127	258374	0.80	0.03	olfactory receptor 127
4933408N05Rik	71122	0.80	0.03	RIKEN cDNA 4933408N05 gene
4930547E14Rik	75145	0.80	0.01	RIKEN cDNA 4930547E14 gene
Dhrs1	52585	0.80	0.00	dehydrogenase/reductase (SDR family) member 1
Bcat2	12036	0.80	0.01	branched chain aminotransferase 2, mitochondrial
1700012J22Rik	75496	0.80	0.04	RIKEN cDNA 1700012J22 gene
Efcbp2	117148	0.80	0.00	EF hand calcium binding protein 2
Rab23	19335	0.80	0.02	RAB23, member RAS oncogene family
Gpld1	14756	0.80	0.00	glycosylphosphatidylinositol specific phospholipase D1
Tomm40	53333	0.80	0.05	translocase of outer mitochondrial membrane 40 homolog (yeast)
Tln1	21894	0.80	0.01	talin 1
Tsc22d4	78829	0.80	0.01	TSC22 domain family 4
Dnahc1	110084	0.80	0.03	dynein, axonemal, heavy chain 1
1700128I11Rik	73615	0.80	0.03	RIKEN cDNA 1700128I11 gene
Tcf4	21413	0.80	0.01	transcription factor 4

A p-value <0.05 indicates that the expression ratio differed significantly from 1.

The changes for 23 strongly up- and down-regulated genes (according to the microarray analysis) were validated by RT-qPCR analysis (Table 5). The gene expression values (Ct) did not depend significantly on the type of muscle (the tibialis anterior vs. the soleus) but there was a statistically significant interaction with the EPO-d treatment factor. There was a significant Pearson correlation of 0.87 (p < 0.01) for the expression values of 19 genes tested by the two methods RT-qPCR and microarray analysis, respectively. Despite the differences in methodologies, gene probes and primers, the regulation results (up- or down-regulation) were consistent for 10 genes. Two genomic markers of hypoxia (hypoxia-inducible factor 1 alpha subunit (Hif1a) and vascular endothelial growth factor alpha (VEGF-alpha)) were significantly up-regulated in RT-qPCR but not in the microarray analysis after filtering for the p-value (slight up-regulation (1 < normalized ratio <1.2); p = 0.21 and 0.22, respectively.

**Table 5:** RT-qPCR results for 23 genes that were up- or down-regulated according to the microarray analysis

**Detector**	**Ct Epo-d mice**	**Ct control mice**	**2-ddCt (fold change)**	**Microarray normalized ratio**
Serpinb2	26.13	12.84	9.90E-05	0.57
Nrap	24.00	12.64	3.76E-04	0.63
Chfr	19.55	14.23	2.48E-02	1.71
Plcb2	20.42	16.01	4.66E-02	1.87
Chac1	15.50	11.66	6.93E-02	0.6
Ctsd	16.82	13.12	7.68E-02	0.66
Pip4k2b	15.07	12.43	0.16	1.70
Runx1	19.07	17.73	0.39	1.88
Eno3	13.69	12.71	0.51	0.53
Mcptl	13.48	12.63	0.55	3.25
Atp1a2	12.90	12.62	0.82	0.66
Hspb7	12.67	12.78	1.08	0.58
Socs3	15.95	16.60	1.55	0.88
Ccdc130	12.38	13.31	1.90	1.74
Isg20l2	12.45	13.61	2.22	1.70
Ckm	12.26	13.58	2.47	0.52
Ints1	18.37	19.98	3.03	1.71
Zp2	10.82	12.74	3.76	0.65
Erk1	14.33	24.29	982.97	1.11
Epor	13.83	12.85	0.50	NA
Cish2	12.89	12.90	1.00	NA
Vegf	13.04	13.89	1.79	NA
Hif1a	14.08	19.84	53.82	NA

The averaged Ct values of the triplicate analysis are quoted for EPO-deficient and control mice. The Ct values were normalized against Cish2 expression because the latter was not affected by the EPO deficiency. The relative expression of each gene in EPO-deficient mice was calculated by comparison with the corresponding gene expression in control mice. There was a statistically significant correlation (Pearson coefficient: 0.87; p < 0.01) between the expression values for 19 genes according to the RT-qPCR assays and the expression values according to the microarray analysis.

### Gene functions and pathways

We used Ingenuity Pathway Analysis® to classify our list of modulated genes by cellular function and by canonical and signaling pathway. Three groups of functions were detected (Table 6): (i) catabolic pathways, including cell death, protein degradation and lipid metabolism, (ii) anabolic pathways, including protein synthesis, cell cycle, growth and proliferation, DNA replication, cellular assembly and molecular transport; (iii) communication, with cell-to-cell signaling and cell signaling. Cell death, protein synthesis, cellular growth and proliferation and lipid metabolism were the main cell functions that were significant associated with between 9 and 12 modulated genes (Table 6).

**Table 6:** Classification of the significantly modulated genes according to their involvement in cell functions or pathways

**Ontology category**	**Number of genes**	**P-value**	**Gene lists**
Cell Death	12	3.47E-05 - 4.53E-02	METAP2 (includes EG:10988), BACE1, ACE, CHFR, MIF, ATP1A2, SERPINB2, CLN3, RUNX1, GMFG, XRCC6, CTSD
Protein Synthesis	11	1.77E-05 - 4.53E-02	TRNY, METAP2 (includes EG:10988), TRNN, ACE, BACE1, CHFR, MIF, CLN3, TRND, UBE2G1, CTSD
Cellular Growth and Proliferation	9	1.06E-03 - 4.53E-02	PIP4K2B, METAP2 (includes EG:10988), CHFR, MIF, CLN3, SERPINB2, RUNX1, CTSD, XRCC6
Lipid Metabolism	9	2.41E-03 - 4.53E-02	PIP4K2B, DHRS4, ALDH8A1, CYGB, ACE, MIF, CLN3, RUNX1, PLCB2
Molecular Transport	8	5.13E-03 - 4.53E-02	ALDH8A1, ACE, MIF, CLN3, ATP1A2, RUNX1, PLCB2, CKM
Cellular Assembly and Organization	7	2.57E-03 - 4.53E-02	METAP2 (includes EG:10988), ACE, BACE1, CLN3, CKM, XRCC6, CTSD
Protein Degradation	6	1.31E-04 - 5.4E-03	ACE, BACE1, CHFR, CLN3, UBE2G1, CTSD
Cardiovascular System Development and Function	5	1.43E-03 - 4.56E-02	METAP2 (includes EG:10988), ACE, MIF, ATP1A2, RUNX1
DNA Replication, Recombination, and Repair	5	7.69E-03 - 4.78E-02	METAP2 (includes EG:10988), CHFR, MIF, ATP1A2, XRCC6
Hematological System Development and Function	4	1.06E-03 - 4.04E-02	ACE, MIF, SERPINB2, RUNX1
Cell-To-Cell Signaling and Interaction	4	1.3E-03 - 4.53E-02	METAP2 (includes EG:10988), ACE, MIF, ZP2
Cell Cycle	4	2.57E-03 - 4.78E-02	METAP2 (includes EG:10988), CHFR, MIF, RUNX1
Cell Signaling	2	1.28E-02 - 2.29E-02	PLCB2, CKM

**Figure 2:**
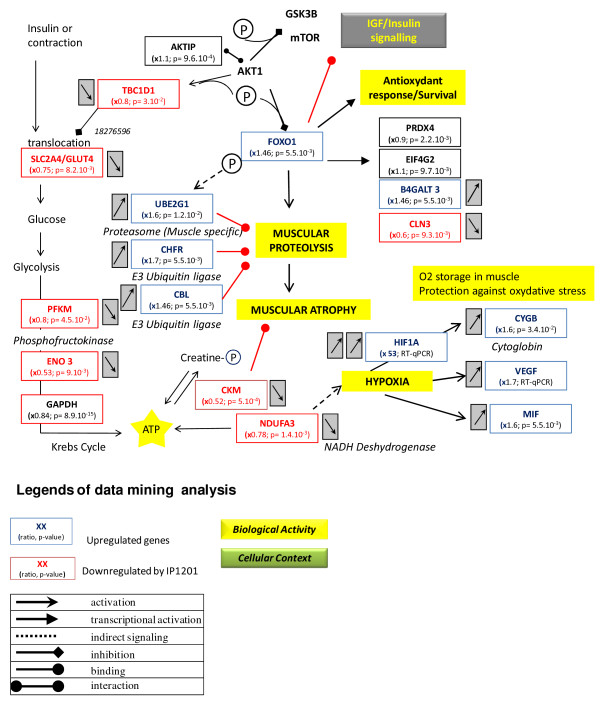
**Main pathway disorders leading to muscle proteolysis, atrophy, hypoxia and anti-oxidant response.** Legends of the figures 2- 5 are indicated under this figure.

**Figure 3:**
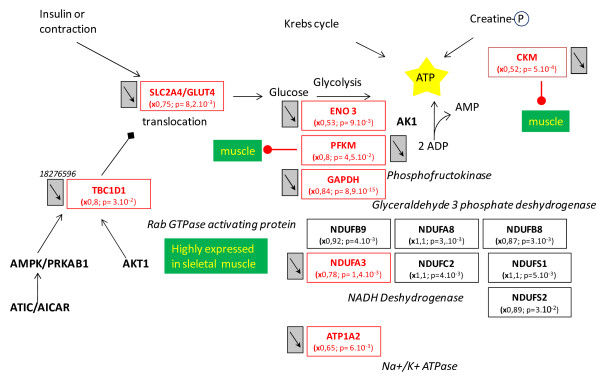
**Impairment in ATP production. Impaired ATP production, with down-regulation of genes involved in glycolysis, mitochondrial oxidative phosphorylation and the phosphocreatine kinase pathway.** Other than some moderately up- or down-regulated subunits of NADH dehydrogenase, most of the genes were down-regulated. Legends of this figure are provided in Figure 2.

**Figure 4:**
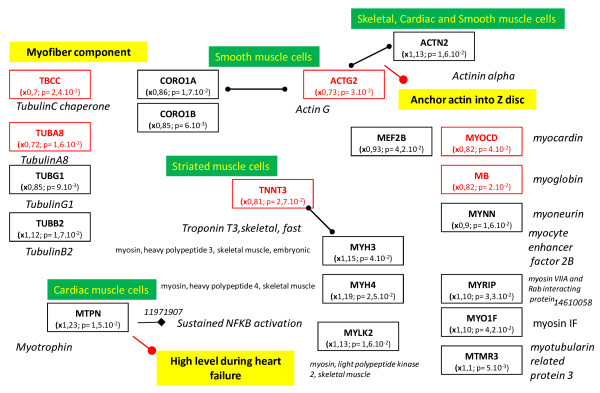
**The gene regulation of cytoskeleton components.** None of the genes were significantly up-regulated (x > 1.4) but seven were moderately up-regulated ( 1 > x > 1.4). All other genes in this category were moderately or severely down-regulated. Legends of this figure are provided in Figure 2.

**Figure 5:**
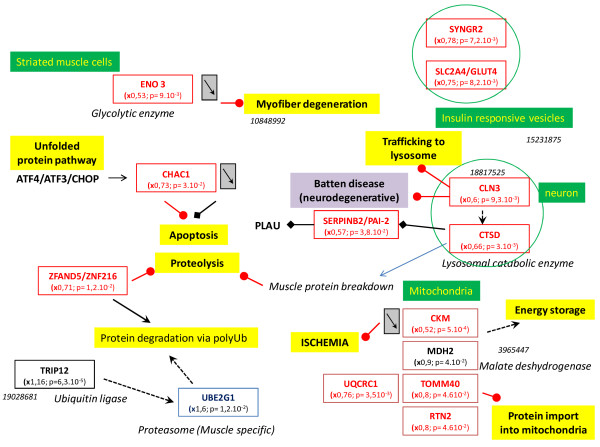
**Down-regulation of many genes involved in intracellular trafficking.** Legends of this figure are provided in Figure 2.

The tissue, organ and cellular context and the associated biological activities were further investigated with a Predictsearch® analysis. Using the PubMed database, it was found that 31 of the significantly modulated genes were strongly associated with the following biological activities: apoptosis, proteolysis, glycolysis, cellular trafficking and cytoskeleton. In addition, the most frequently cited disease pathways in relation to the gene set were muscle atrophy, hypoxia and oxidative stress. The main pathway disruptions revealed by Predictsearch® data mining are described in Figures 2, 3, 4 and 5.

The forkhead box O1 (Foxo1) gene was significantly over-expressed and may activate muscle proteolysis in conjunction with three other up-regulated genes involved in the muscle proteasome (Ube2g1) and E3 Ubiquitin ligase (Chfr and Cbl) (Figure 2). Chronic proteolysis of myofibrils may be responsible for muscle atrophy. Glucose import into muscle cells by the glucose transporter type 4, insulin-responsive (Slc2a4/Glut4), was down-regulated and probably contributed to a decrease in ATP production. In addition, several genes coding for key enzymes in glycolysis (Pfkm, Eno3 and Gapdh), mitochondrial oxidative phosphorylation (NADH dehydrogenase unit Ndufa3) and the muscle creatine kinase (Ckm) were down-regulated. The two genomic markers of muscle hypoxia (Hif1a and Vegf) were up-regulated. Low mitochondrial activity seemed to be related to hypoxia signaling with two up-regulated downstream genes: cytoglobin (Cygb) and macrophage migration inhibitory factor (Mif). The up-regulation of Foxo1 gene could be interpreted as a signal of anti-oxidant response by the muscle cells, with up-regulation of glycoprotein-4-beta-galactosyltransferase 2 (B4galt) and eukaryotic translation initiation factor 4 gamma (Elf4g2) and down-regulation of ceroid lipofuscinosis neuronal 3 (Cln3).

Seven genes coding for cytoskeleton components were moderately up-regulated (1 < x < 1.4) and all the other genes were moderately or significantly down-regulated (Figure 4). These down-regulations may affect the synthesis of the cytoskeleton proteins: actin, tubulin, troponin and myosin. Decreased expression of the genes coding for cytoskeleton components and ATP production by glycolysis may be related to the decrease in intracellular trafficking (Figure 5). Mitochondrial protein import was probably decreased by the concomitant down-regulation of five genes: Tomm40, Ckm, Mdh2, Rtn2 and Uqcr1. These changes may also have contributed to the decrease in mitochondrial oxidative phosphorylation activity. The insulin-responsive vesicles transporting glucose into the cells (Sclc2a4/Glut4; Syngr2) and lysosomal vesicles transporting catabolic enzymes (Cln3 and Ctsd) were significantly down-regulated. Protein degradation in the proteasome was stimulated by the up-regulated ubiquitin-conjugating enzyme (Ube2g1) and the down-regulated zinc finger protein (Zfand5/Znf216). Down-regulation of the cation transport regulator-like protein 1 (Chac1) may contribute to the inhibition of apoptosis. The cell survival factor serine (or cysteine or plasminogen) peptidase inhibitor (Serpinb2/Pai-2) was strongly down-regulated and would not have provided its usual cell protection activity.

## Discussion

Most of the studies on muscle exercise or doping are based on the use of acute or chronic EPO injection to stimulate endurance capacity. In the present study, the EPO-d model was used to study the effects of EPO loss-of-function on the muscles of exercising mice. To date, the study of adult EPO-d animals has been limited by the fact that EPO gene knock-out (KO) is embryonic lethal [35,36]. Protein functional knock-out by immunoneutralization of circulating EPO is a novel way of inducing EPO deficiency in live, active mice [27]. Thus, the effects of the EPO deficiency were evaluated through an integrative biology approach in order to reveal potential consequences at the physiologic and genomic levels. The EPO-d mice had an Htc of about 20%, i.e. two-fold lower than in control mice and much lower than the value of 58% for maximal exercise performance in wild-type mice [37]. However, this low Htc did not prevent the performance of valid exercise tests. Another type of murine EPO-d model is the EPO-Tag^h^ transgenic mouse, which displayed about the same, low Htc (19.3%) in normoxia as in our model [38]. After two weeks of rest, the researchers did not observe an elevation in Hif1a or muscle atrophy - suggesting that a lack of EPO does not cause structural damage to muscles. However, the studied animals did not perform exercise. In fact, the observed activation of the EPO-R by acute exercise suggests that physiological levels of EPO do have a role in exercising muscle tissue [12]. Indeed, the combination of exercise and chronic EPO deficiency in the present study resulted in hypoxia with Hif1a accumulation and muscle atrophy.

In the present study, reductions of between 25% and 30% in vPeak, VO_2max_, and CS attested to the subnormal exercise performance in EPO-d mice. However, the time to exhaustion was not different between groups. This is not consistent with the 50% decrease (vs. controls) in the time to exhaustion in a swimming test observed in a murine CKF model with a low Htc (28% lower than in controls). However, the intensity of the swimming exercise was not quantified and the absolute intensity was probably similar in the two groups. In the present study, performance was first assessed in terms of vPeak and VO_2max_. Next, the intensity chosen for the exhaustive exercise (at CS) was individually related to performance (at around 80% of vPeak); thus, all mice were exercising at the same relative intensity. This explained the fact that the time to exhaustion was similar in the two groups and confirmed the relevance of CS in the running mouse model [30]. Furthermore, VO_2max_ was correlated with the Htc as previously observed by Schuler (2010). This relation is valid in each group and in the whole study population. These results validate this EPO-d mouse model in physiological terms. However, in the present study, total hemoglobin was not determined. This question is controversial; in general, total hemoglobin is correlated with VO_2max_ rather than Htc. Indeed, elevated hemoglobin is correlated with an elevated VO_2max_ in humans [39] and there is an optimal Htc (57%-58%) for a maximal performance in EPO-treated mice [37]. Above this level, performance levels decrease and excessive erythrocytosis leads to multiple organ degeneration, including damage to heart and skeletal muscles [40,41]. The impact of hemoglobin concentration on VO_2max_ becomes greater under non-physiological conditions (such as blood loss or doping). Indeed, data from anti-doping studies generally show a similar relationship between the hemoglobin concentration and VO_2max_, as observed after blood loss [39]. However, in contrast with data from humans, the control mice in the present study showed a significant correlation between Htc vs. VO_2max_. (*i.e.* in the physiological range).

This set of physiological results emphasized the poor endurance capacity in EPO-d mice, which may be related to their impaired aerobic capacity relative to control mice. They exhibited a lower VO_2max_ and higher blood [La]_INC_ which are physiological signs of hypoxia. This was confirmed at the genomic level by strong up-regulation of Hif1a, which acts as the master regulator for the expression of genes involved in the hypoxia response in most mammalian cells [42,43]. There were some indirect signs of muscle hypoxia, with the up-regulation of Vegf, Cygb and Mif. In addition, some NADH sub-units involved in mitochondrial oxidative phosphorylation and other genes coding for mitochondrial proteins (Ckm, Mdh2, Tomm40, Rtn2, Uqcrc1) were down-regulated. Chronic muscle hypoxia could be directly related to the animals’ low Htc and the drop of oxygen tension in the muscle fibers and mitochondria. This hypoxia was associated with cell oxidative stress, as indicated by the up-regulation of the Foxo1 marker. On the mitochondrial level, it has been reported that about 2% of the oxygen used by the respiratory chain is incompletely reduced and produces superoxide radicals [44]. The overproduction of reactive oxygen species (ROS) in hypoxia is a surprising finding but has been previously described in hypoxic cardiomyocytes [45] and in muscles with glycogenosis and mitochondrial dysfunction [46]. In the present study, we found that several genes coding for NADH subunits were down-regulated, resulting in uncoupling with the Krebs cycle. Thus, the respiratory chain complex I could be responsible of the lack of reduction and would provide electrons for ROS generation. If O_2_ is not entirely reduced to water by complex IV, ROS generation seems to contribute to the O_2_ signaling pathway [47]. The ROS-like superoxide anion radical O_2_^-^, hydrogen peroxide and the hydroxyl radical .OH are all able to damage proteins, DNA and membranes. However, the generation of .OH from hydrogen peroxide by an iron-dependent Fenton reaction within or close to the nucleus could trigger the expression of redox-sensitive transcription factors such as Hif1a. Thus, ROS generation could be the primary messenger responsible for Hif1 transcription factor regulation, which may explain the relationship between oxidative stress and hypoxia.

The present transcriptome data mining analysis showed that the EPO deficiency induced expression changes in genes related to muscle hypoxia and proteolysis. In addition, 12 significantly modulated genes were involved in cell death and apoptosis (Table 6). Thus, one can assume that physiological concentrations of circulating EPO have a muscle-protecting effect. It has been reported that after acute exercise, EPO-Rs are up-regulated in satellite cells and muscle fibers - suggesting a muscle-specific EPO signaling pathway [12]. The muscle-protecting effect of EPO could be related to anti-apoptotic activity, as has been demonstrated in a model of cadiomyocyte ischemia [48]. In fact, EPO activates (through the EPO-R) the PI3 kinase /Akt signaling pathway and thus increases both eNOS expression and phosphorylation. Production of NO by eNOS inhibits cardiomyocyte apoptosis. The NO has both anti-apoptotic and pro-apoptotic activities by modulating multiple sites in the death signaling pathway and the mitochondrial apoptosis pathway [49]. However, most of the anti-apoptotic activity seems to occur via an increase in Bcl-2 expression and prevention of cytochrome c release from the mitochondria. Other pathways may have a muscle-protective effect, such as L-type calcium channel inhibition and the up-regulation of protective proteins (such as heme-oxygenase-1, heat shock proteins Hsp70 and metallothionein).

**Figure 6:**
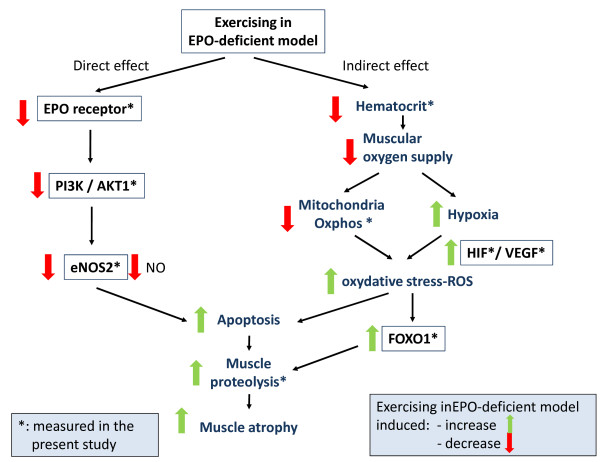
**Overview of the direct and indirect effects of EPO loss-of-function on muscle.** On the basis of the present study's results, our model seeks to explain the impact of a lack of functional EPO on muscle tissue in the EPO-d mouse.

In the present study, some results were consistent with a potential role of EPO in the anti-apoptotic pathway in exercising muscle. We found that EPO-R, nitric oxide synthase 2 inducible (Nos2) and Akt1 were down-regulated in EPO-d mice. Akt is also associated with muscle protein synthesis and hypertrophy [50]. In contrast, EPO injections do not affect EPO-R signaling in muscles under resting conditions - suggesting an indirect effect of EPO related to an increase in oxidative capacity [51]. We did not detect Hsp70 modulation but two Hsp90 genes (Hsp90aa1, Hsp90b1) were up-regulated. The heat shock protein 90 family is known to stabilize and accumulate Hif1a under hypoxic conditions, which is consistent with our observation in an RT-qPCR that Hif1a was highly up-regulated [52]. It has been reported that EPO may also exert an anti-oxidant effect in the blood vessels by up-regulating superoxide dismutase (Sod1) [53]. These previous results may be related to our finding of muscle oxidative stress in EPO-d mice, with a combination of down-regulation of Sod1 and up-regulation of Foxo1 as a response to oxidative stress. Low muscle oxidative stress stimulates muscle adaptation and performance levels but high oxidative stress decreases muscle force production by inhibiting the sarcoplasmic calcium ATPase (Serca) [54], which may explain the poor exercise performance of the EPO-d mice in the present study. In addition, it has been shown that oxidative stress contributes to the increase in muscle atrophy by accelerating muscle protein degradation by the calpains and caspase-3 [55]. However, this observation was made in isolated muscle fibers *in vitro* and one can legitimately suppose that exercise activity may increase this phenomenon, since we observed major proteolysis activity in the present transcriptome analysis. Taken as a whole, our present results and literature data prompted us to suggest a putative signaling pathway that may explain EPO's direct and indirect protective effects on skeletal muscle (Figure 6). Moreover, the transgenic rescued EPO-R-null mutant mice would be an interesting tool for elucidating the relative contributions of EPO's various non-hematopoietic effects [56]. 

## Conclusion

From a physiological point of view, our study validated the EPO-d mouse model by demonstrating worse exercise performance than in control mice with a normal Htc. This poor exercise performance could be explained by the lower Htc, chronic hypoxia, muscle oxidative stress and the severe muscular proteolysis revealed by the transcriptome analysis. Our results suggest that physiological levels of EPO cytokine exert both direct and indirect protective effects on muscle during exercise. However, the signaling pathway involved in these protective effects remains to be described in detail.

## Abbreviations

[La]_INC_Blood lactate concentration at the end of the incremental protocol[La]_SS_Blood lactate concentration at the end of the steady-state protocolaaRNAAmino-allyl RNAAkt1Protein kinase BB4galtbeta-1,4-galactosyltransferaseBcl2B-cell lymphoma 2CbtCabutChac1Cation transport regulator homolog 1CHFChronic heart failureChfrCheckpoint with fork-head associated and ring fingerCKFChronic kidney failureCkmMuscle creatine kinaseCln3Juvenile neuronal ceroid lipofuscinosisCSCritical speedCtCycle thresholdCTSDCathepsin DCYGBCytoglobinDTTDithiothreitolE1f4g2Eno3, enolase 3eNOSEndothelial nitric oxide synthaseEPOErythropoietinEPO-dErythropoietin-deficientEPO-RErythropoietin receptorFoxo1Forkhead box protein O1Hif1aHypoxia-inducible factor 1 alphaHsp70Heat shock protein 70HtcHematocritMdh2Malate dehydrogenase 2MIFMacrophage migration inhibitory factorNos2Nitric oxide synthase 2Pi3kPhosphatidylinositol 3-kinasesrHuEpoRecombinant human erythropoietinROSReactive oxygen speciesRtn2Reticulon 2Serpinb2/Pai2Syngr2, synaptogyrin 2TlimTime limitTomm40Translocase of outer mitochondrial membrane 40 homologUbe2g1Ubiquitin-conjugating enzyme E2 G1VEGFVascular endothelial growth factorvPeakPeak velocity $\dot{\text{V}}\text{O}2$_max_: maximal oxygen consumption; $\dot{\text{V}}\text{O}2$: oxygen consumption; Zfand5/Znf216: Zinc finger A20 domain-containing protein 2.

## Competing interests

The authors declare that they have no competing interests.

## Author's contributions

LH, EB, VB designed the study and drafted the manuscript. LH, EH carried out the mouse tests and physiological analysis. EB, BB carried out the transcriptomic analysis. EH, EB carried out the qPCR validation. FJ, PB carried out the transcriptomic interpretation. All authors read and approved the final manuscript.

## Pre-publication history

The pre-publication history for this paper can be accessed here:

http://www.biomedcentral.com/1755-8794/5/29/prepub

## Supplementary Material

Additional file 1 **Table S1.** Title: List of all significantly modulated genes. Description of data: List of all the significantly modulated genes (p < 0.05) expressed in the tibialis and soleus muscles of EPO-deficient and control mice. The p-value (p < 0.05) indicates that the expression ratio differed significantly from 1. Click here for file
