# Supplementary material for: Skeletal muscle alterations and exercise performance decrease in erythropoietin-deficient mice: a comparative study
Source: BMC Med Genomics. 2012 Jun 29;5:29. doi: 10.1186/1755-8794-5-29 (PMC3473259; doi:10.1186/1755-8794-5-29)
Supplement: Additional file 1 — Table S1. Title: List of all significantly modulated genes. Description of data: List of all the significantly modulated genes (p < 0.05) expressed in the tibialis and soleus muscles of EPO-deficient and control mice. The p-value (p < 0.05) indicates that the expression ratio differed significantly from 1. [file 1755-8794-5-29-S1.doc]

| **Genes** | **Entrez Gene ID** | | **Normalized ratio** | | | **t-testP-value** | | | **Description** | | | | |  |
| --- | --- | --- | --- | --- | --- | --- | --- | --- | --- | --- | --- | --- | --- | --- |
| UP-REGULATEDGENES | |  | | |  | | |  | |  | | | | |
| Mcptl | 17233 | | 3,25 | | | 0,01 | | | mastcellprotease-like | | | | |  |
| Dhrs4 | 28200 | | 2,55 | | | 0,01 | | | dehydrogenase/reductase(SDRfamily)member4 | | | | |  |
| Runx1 | 12394 | | 1,88 | | | 0,03 | | | runtrelatedtranscriptionfactor1 | | | | |  |
| 3830408D24Rik | 100039781 | | 1,87 | | | 0,01 | | | RIKENcDNA3830408D24gene | | | | |  |
| Plcb2 | 18796 | | 1,87 | | | 0,04 | | | phospholipaseC,beta2 | | | | |  |
| 1810013L24Rik | 69053 | | 1,79 | | | 0,05 | | | RIKENcDNA1810013L24gene | | | | |  |
| Aldh8a1 | 237320 | | 1,78 | | | 0,02 | | | aldehydedehydrogenase8family,memberA1 | | | | |  |
| Asp-ARNt_TRND | 4555 | | 1,76 | | | 0,01 | | | mitochondriallyencodedtRNAasparticacid[Homosapiens] | | | | |  |
| Ccdc130 | 67736 | | 1,74 | | | 0,02 | | | coiled-coildomaincontaining130 | | | | |  |
| Chfr | 231600 | | 1,71 | | | 0,02 | | | checkpointwithforkheadandringfingerdomains | | | | |  |
| Ints1 | 68510 | | 1,71 | | | 0,02 | | | integratorcomplexsubunit1 | | | | |  |
| Pip5k2b | 108083 | | 1,70 | | | 0,02 | | | phosphatidylinositol-5-phosphate4-kinase,typeII,beta | | | | |  |
| Isg20l2 | 229504 | | 1,70 | | | 0,01 | | | interferonstimulatedexonucleasegene20-like2 | | | | |  |
| Bace1 | 23821 | | 1,69 | | | 0,04 | | | beta-siteAPPcleavingenzyme1 | | | | |  |
| 1200015F23Rik | 67809 | | 1,68 | | | 0,02 | | | RIKENcDNA1200015F23gene | | | | |  |
| Gmfg | 63986 | | 1,67 | | | 0,02 | | | gliamaturationfactor,gamma | | | | |  |
| BC037032 | 414066 | | 1,65 | | | 0,01 | | | cDNASequenceBC037032 | | | | |  |
| Zdhhc9 | 208884 | | 1,65 | | | 0,03 | | | zincfinger,DHHCdomaincontaining9 | | | | |  |
| Metap2 | 56307 | | 1,62 | | | 0,02 | | | methionineaminopeptidase2 | | | | |  |
| 1110008L16Rik | 66132 | | 1,61 | | | 0,03 | | | RIKENcDNA1110008L16gene | | | | |  |
| Asn-ARNt_TRNN | 4570 | | 1,61 | | | 0,00 | | | mitochondriallyencodedtRNAasparagine[Homosapiens] | | | | |  |
| Ube2g1 | 67128 | | 1,61 | | | 0,01 | | | ubiquitin-conjugatingenzymeE2G1(UBC7homolog,C.elegans) | | | | |  |
| 4933409D19Rik | 71084 | | 1,61 | | | 0,05 | | | RIKENcDNA4933409D19gene | | | | |  |
| Tyr-ARNt_TRNY | 4579 | | 1,60 | | | 0,03 | | | mitochondrially encoded tRNA tyrosine | | | | |  |
| Mif | 17319 | | 1,60 | | | 0,04 | | | macrophagemigrationinhibitoryfactor | | | | |  |
| Olfr1428 | 258673 | | 1,60 | | | 0,03 | | | olfactoryreceptor1428 | | | | |  |
| Cygb | 114886 | | 1,60 | | | 0,03 | | | cytoglobin | | | | |  |
| Olfr1387 | 258465 | | 1,59 | | | 0,01 | | | olfactoryreceptor1387 | | | | |  |
| Adarb1 | 110532 | | 1,58 | | | 0,01 | | | adenosinedeaminase,RNA-specific,B1 | | | | |  |
| BC023105 | 207269 | | 1,58 | | | 0,02 | | | cDNAsequenceBC023105 | | | | |  |
| Aak1 | 269774 | | 1,56 | | | 0,02 | | | AP2associatedkinase1 | | | | |  |
| 9830134C10Rik | 442827 | | 1,56 | | | 0,00 | | | RIKENcDNA9830134C10gene | | | | |  |
| 4921530L21Rik | 66732 | | 1,55 | | | 0,04 | | | RIKENcDNA4921530L21gene | | | | |  |
| A930014E01Rik | 78598 | | 1,52 | | | 0,03 | | | RIKENcDNAA930014E01gene | | | | |  |
| Dgkh | 380921 | | 1,51 | | | 0,04 | | | diacylglycerolkinase,eta | | | | |  |
| Ccdc72 | 66167 | | 1,51 | | | 0,05 | | | coiled-coildomaincontaining72 | | | | |  |
| V1rc1 | 113858 | | 1,51 | | | 0,00 | | | vomeronasal1receptor,C1 | | | | |  |
| Zim1 | 22776 | | 1,51 | | | 0,01 | | | zincfinger,imprinted1 | | | | |  |
| Ace | 11421 | | 1,50 | | | 0,04 | | | angiotensinIconvertingenzyme(peptidyl-dipeptidaseA)1 | | | | |  |
| Xrcc6 | 14375 | | 1,50 | | | 0,01 | | | X-rayrepaircomplementingdefectiverepairinChinesehamstercells6 | | | | |  |
| Dcun1d3 | 233805 | | 1,50 | | | 0,02 | | | DCN1,defectiveincullinneddylation1,domaincontaining3(S.cerevisiae) | | | | |  |
| Adam28 | 13522 | | 1,49 | | | 0,04 | | | adisintegrinandmetallopeptidasedomain28 | | | | |  |
| Adamts10 | 224697 | | 1,49 | | | 0,03 | | | adisintegrin-likeandmetallopeptidase(reprolysintype)withthrombospondintype1motif,10 | | | | |  |
| Bach2 | 12014 | | 1,46 | | | 0,03 | | | BTBandCNChomology2 | | | | |  |
| Cbl | 12402 | | 1,46 | | | 0,01 | | | CasitasB-lineagelymphoma | | | | |  |
| Hist1h2an | 319170 | | 1,46 | | | 0,02 | | | histonecluster1,H2an | | | | |  |
| Eif5a | 276770 | | 1,45 | | | 0,05 | | | eukaryotictranslationinitiationfactor5A | | | | |  |
| Zcchc2 | 227449 | | 1,45 | | | 0,02 | | | zincfinger,CCHCdomaincontaining2 | | | | |  |
| Stk24 | 223255 | | 1,45 | | | 0,05 | | | serine/threoninekinase24(STE20homolog,yeast) | | | | |  |
| B4galt3 | 57370 | | 1,45 | | | 0,02 | | | UDP-Gal:betaGlcNAcbeta1,4-galactosyltransferase,polypeptide3 | | | | |  |
| Tprkb | 69786 | | 1,44 | | | 0,00 | | | Tp53rkbindingprotein | | | | |  |
| Sfpq | 71514 | | 1,44 | | | 0,05 | | | splicingfactorproline/glutaminerich(polypyrimidinetractbindingproteinassociated) | | | | |  |
| Foxo1 | 56458 | | 1,44 | | | 0,02 | | | forkheadboxO1 | | | | |  |
| 4933436I01Rik | 66780 | | 1,44 | | | 0,03 | | | RIKENcDNA4933436I01gene | | | | |  |
| 9830107B12Rik | 328829 | | 1,43 | | | 0,01 | | | RIKENcDNA9830107B12gene | | | | |  |
| Dhx30 | 72831 | | 1,43 | | | 0,02 | | | DEAH(Asp-Glu-Ala-His)boxpolypeptide30 | | | | |  |
| Sema3e | 20349 | | 1,43 | | | 0,01 | | | semadomain,immunoglobulindomain(Ig),shortbasicdomain,secreted,(semaphorin)3E | | | | |  |
| Vps37b | 330192 | | 1,42 | | | 0,03 | | | vacuolarproteinsorting37B(yeast) | | | | |  |
| AI662250 | 106639 | | 1,41 | | | 0,05 | | | expressedsequenceAI662250 | | | | |  |
| Arhgap26 | 71302 | | 1,41 | | | 0,01 | | | RhoGTPaseactivatingprotein26 | | | | |  |
| 4930434E21Rik | 381693 | | 1,41 | | | 0,04 | | | RIKENcDNA4930434E21gene | | | | |  |
| 1500001A10Rik | 68955 | | 1,41 | | | 0,02 | | | RIKENcDNA1500001A10gene | | | | |  |
| Prtg | 235472 | | 1,40 | | | 0,00 | | | protogeninhomolog(Gallusgallus) | | | | |  |
| Lefty2 | 320202 | | 1,40 | | | 0,02 | | | Left-rightdeterminationfactor2 | | | | |  |
| 4933431J24Rik | 71298 | | 1,40 | | | 0,00 | | | RIKENcDNA4933431J24gene | | | | |  |
| Opn1sw | 12057 | | 1,40 | | | 0,01 | | | opsin1(conepigments),short-wave-sensitive(colorblindness,tritan) | | | | |  |
| Vcpip1 | 70675 | | 1,40 | | | 0,01 | | | valosincontainingprotein(p97)/p47complexinteractingprotein1 | | | | |  |
| Defb37 | 353320 | | 1,40 | | | 0,01 | | | defensinbeta37 | | | | |  |
| LOW-MODULATEDGENES | | | |  | | |  | | | |  |  |  | |
| Ccrk | 105278 | | 1,39 | | | 0,03 | | | cellcyclerelatedkinase | | | | |  |
| 4930579P08Rik | 75878 | | 1,39 | | | 0,02 | | | RIKENcDNA4930579P08gene | | | | |  |
| Armet | 74840 | | 1,39 | | | 0,02 | | | arginine-rich,mutatedinearlystagetumors | | | | |  |
| Ctsk | 13038 | | 1,38 | | | 0,01 | | | cathepsinK | | | | |  |
| 4930548J01Rik | 78043 | | 1,38 | | | 0,02 | | | RIKENcDNA4930548J01gene | | | | |  |
| 4833409A17Rik | 74580 | | 1,38 | | | 0,01 | | | RIKENcDNA4833409A17gene | | | | |  |
| 9530086P17Rik | 78742 | | 1,38 | | | 0,00 | | | RIKENcDNA9530086P17gene | | | | |  |
| 4930471E15Rik | 75020 | | 1,38 | | | 0,02 | | | RIKENcDNA4930471E15gene | | | | |  |
| Clcn5 | 12728 | | 1,38 | | | 0,04 | | | chloridechannel5 | | | | |  |
| BC037438 | 403345 | | 1,37 | | | 0,01 | | | cDNAsequenceBC037438 | | | | |  |
| Adam39 | 546055 | | 1,37 | | | 0,05 | | | adisintegrinandmetallopeptidasedomain39 | | | | |  |
| D030070L09Rik | 225280 | | 1,37 | | | 0,03 | | | RIKENcDNAD030070L09gene | | | | |  |
| Ccr1 | 12768 | | 1,37 | | | 0,02 | | | chemokine(C-Cmotif)receptor1 | | | | |  |
| Olfr1079 | 258402 | | 1,37 | | | 0,02 | | | olfactoryreceptor1079 | | | | |  |
| Hnrpll | 72692 | | 1,37 | | | 0,04 | | | heterogeneousnuclearribonucleoproteinL-like | | | | |  |
| A330102K18Rik | 77919 | | 1,37 | | | 0,03 | | | RIKENcDNAA330102K18gene | | | | |  |
| MMP13 | 4322 | | 1,36 | | | 0,05 | | |  | | | | |  |
| Trim36 | 28105 | | 1,36 | | | 0,05 | | | tripartitemotif-containing36 | | | | |  |
| Cttnbp2nl | 80281 | | 1,36 | | | 0,02 | | | CTTNBP2N-terminallike | | | | |  |
| Tom1l1 | 71943 | | 1,36 | | | 0,03 | | | targetofmyb1-like1(chicken) | | | | |  |
| E330017A01Rik | 224247 | | 1,35 | | | 0,02 | | | RIKENcDNAE330017A01gene | | | | |  |
| Kif3a | 16568 | | 1,35 | | | 0,04 | | | kinesinfamilymember3A | | | | |  |
| Cacng4 | 54377 | | 1,35 | | | 0,01 | | | calciumchannel,voltage-dependent,gammasubunit4 | | | | |  |
| 6230409E13Rik | 76132 | | 1,35 | | | 0,03 | | | RIKENcDNA6230409E13gene | | | | |  |
| Atg4b | 66615 | | 1,35 | | | 0,01 | | | autophagy-related4B(yeast) | | | | |  |
| Ccdc84 | 382073 | | 1,35 | | | 0,02 | | | coiled-coildomaincontaining84 | | | | |  |
| Cd93 | 17064 | | 1,35 | | | 0,00 | | | CD93antigen | | | | |  |
| Slbp | 20492 | | 1,35 | | | 0,03 | | | stem-loopbindingprotein | | | | |  |
| Bat2d | 226562 | | 1,35 | | | 0,02 | | | BAT2domaincontaining1 | | | | |  |
| Srxn1 | 76650 | | 1,34 | | | 0,02 | | | sulfiredoxin1homolog(S.cerevisiae) | | | | |  |
| 4930483K19Rik | 74953 | | 1,34 | | | 0,03 | | | RIKENcDNA4930483K19gene | | | | |  |
| Olfr702 | 258590 | | 1,34 | | | 0,02 | | | olfactoryreceptor702 | | | | |  |
| Abcb10 | 23456 | | 1,34 | | | 0,02 | | | ATP-bindingcassette,sub-familyB(MDR/TAP),member10[Homosapiens] | | | | |  |
| Map2k2 | 26396 | | 1,34 | | | 0,02 | | | mitogenactivatedproteinkinasekinase2 | | | | |  |
| Olfr1097 | 258840 | | 1,34 | | | 0,02 | | | olfactoryreceptor1097 | | | | |  |
| 4632427E13Rik | 666737 | | 1,33 | | | 0,03 | | | RIKENcDNA4632427E13gene | | | | |  |
| Bst2 | 69550 | | 1,33 | | | 0,03 | | | bonemarrowstromalcellantigen2 | | | | |  |
| 4930422C21Rik | 75781 | | 1,33 | | | 0,03 | | | RIKENcDNA4930422C21gene | | | | |  |
| Igj | 16069 | | 1,33 | | | 0,02 | | | immunoglobulinjoiningchain | | | | |  |
| 9030416H16Rik | 71521 | | 1,33 | | | 0,04 | | | PDS5,regulatorofcohesionmaintenance,homologA(S.cerevisiae) | | | | |  |
| Rab35 | 77407 | | 1,32 | | | 0,02 | | | RAB35,memberRASoncogenefamily | | | | |  |
| Sgk | 20393 | | 1,32 | | | 0,00 | | | serum/glucocorticoidregulatedkinase | | | | |  |
| Olfr1414 | 259041 | | 1,31 | | | 0,03 | | | olfactoryreceptor1414 | | | | |  |
| Col10a1 | 12813 | | 1,31 | | | 0,03 | | | procollagen,typeX,alpha1 | | | | |  |
| 4632428N05Rik | 74048 | | 1,31 | | | 0,04 | | | RIKENcDNA4632428N05gene | | | | |  |
| 1700006J14Rik | 321010 | | 1,31 | | | 0,01 | | | RIKENcDNA1700006J14gene | | | | |  |
| Aldh1a3 | 56847 | | 1,31 | | | 0,03 | | | aldehydedehydrogenasefamily1,subfamilyA3 | | | | |  |
| Igsf1 | 209268 | | 1,31 | | | 0,00 | | | immunoglobulinsuperfamily,member1 | | | | |  |
| Retnla | 57262 | | 1,31 | | | 0,00 | | | resistinlikealpha | | | | |  |
| BC049635 | 277773 | | 1,31 | | | 0,01 | | | cDNAsequenceBC049635 | | | | |  |
| Olfr961 | 258497 | | 1,31 | | | 0,03 | | | olfactoryreceptor961 | | | | |  |
| Selplg | 20345 | | 1,30 | | | 0,02 | | | selectin,platelet(p-selectin)ligand | | | | |  |
| Prssl1 | 73106 | | 1,30 | | | 0,05 | | | protease,serine-like1 | | | | |  |
| 1110014K08Rik | 319370 | | 1,30 | | | 0,03 | | | RIKENcDNA1110014K08gene | | | | |  |
| Bxdc1 | 67239 | | 1,30 | | | 0,00 | | | brixdomaincontaining1 | | | | |  |
| Cdh18 | 320865 | | 1,30 | | | 0,04 | | | cadherin18 | | | | |  |
| Kcne1l | 66240 | | 1,30 | | | 0,04 | | | potassiumvoltage-gatedchannel,Isk-relatedfamily,member1-like | | | | |  |
| Lrguk | 74354 | | 1,30 | | | 0,01 | | | leucine-richrepeatsandguanylatekinasedomaincontaining | | | | |  |
| Synpo | 104027 | | 1,30 | | | 0,02 | | | synaptopodin | | | | |  |
| Impad1 | 242291 | | 1,30 | | | 0,02 | | | inositolmonophosphatasedomaincontaining1 | | | | |  |
| A2bp1 | 268859 | | 1,30 | | | 0,03 | | | ataxin2bindingprotein1 | | | | |  |
| Gli2 | 14633 | | 1,30 | | | 0,03 | | | GLI-KruppelfamilymemberGLI2 | | | | |  |
| Gp1ba | 14723 | | 1,30 | | | 0,02 | | | glycoprotein1b,alphapolypeptide | | | | |  |
| Bat2 | 53761 | | 1,30 | | | 0,05 | | | HLA-Bassociatedtranscript2 | | | | |  |
| 1810012K16Rik | 69108 | | 1,30 | | | 0,04 | | | RIKENcDNA1810012K16gene | | | | |  |
| Fcer2a | 14128 | | 1,30 | | | 0,03 | | | Fcreceptor,IgE,lowaffinityII,alphapolypeptide | | | | |  |
| Hist1h3d | 319149 | | 1,29 | | | 0,02 | | | histonecluster1,H3d | | | | |  |
| Vim | 22352 | | 1,29 | | | 0,00 | | | vimentin | | | | |  |
| 4930545E07Rik | 75162 | | 1,29 | | | 0,01 | | | RIKENcDNA4930545E07gene | | | | |  |
| Acp6 | 66659 | | 1,29 | | | 0,04 | | | acidphosphatase6,lysophosphatidic | | | | |  |
| Ifi44 | 99899 | | 1,29 | | | 0,01 | | | interferon-inducedprotein44 | | | | |  |
| 4930402D18Rik | 74852 | | 1,29 | | | 0,01 | | | RIKENcDNA4930402D18gene | | | | |  |
| 1700040G22Rik | 76714 | | 1,29 | | | 0,01 | | | RIKENcDNA1700040G22gene | | | | |  |
| Col3a1 | 12825 | | 1,29 | | | 0,01 | | | procollagen,typeIII,alpha1 | | | | |  |
| D730047N16Rik | 77988 | | 1,29 | | | 0,05 | | | RIKENcDNAD730047N16gene | | | | |  |
| Slc24a2 | 76376 | | 1,29 | | | 0,02 | | | solutecarrierfamily24(sodium/potassium/calciumexchanger),member2 | | | | |  |
| Lysmd3 | 80289 | | 1,29 | | | 0,02 | | | LysM,putativepeptidoglycan-binding,domaincontaining3 | | | | |  |
| Mmp21 | 214766 | | 1,29 | | | 0,00 | | | matrixmetallopeptidase21 | | | | |  |
| 5730408A14Rik | 70516 | | 1,29 | | | 0,04 | | | RIKENcDNA5730408A14gene | | | | |  |
| Hmgcr | 15357 | | 1,28 | | | 0,00 | | | 3-hydroxy-3-methylglutaryl-CoenzymeAreductase | | | | |  |
| Nudt13 | 67725 | | 1,28 | | | 0,05 | | | nudix(nucleosidediphosphatelinkedmoietyX)-typemotif13 | | | | |  |
| Crb3 | 224912 | | 1,28 | | | 0,04 | | | crumbshomolog3(Drosophila) | | | | |  |
| Prpf6 | 68879 | | 1,28 | | | 0,04 | | | PRP6pre-mRNAsplicingfactor6homolog(yeast) | | | | |  |
| Olfr58 | 18358 | | 1,28 | | | 0,05 | | | olfactoryreceptor58 | | | | |  |
| 4921506J03Rik | 382423 | | 1,28 | | | 0,02 | | | RIKENcDNA4921506J03gene | | | | |  |
| Ndst3 | 83398 | | 1,28 | | | 0,01 | | | N-deacetylase/N-sulfotransferase(heparanglucosaminyl)3 | | | | |  |
| 6820431F20Rik | 381598 | | 1,28 | | | 0,00 | | | RIKENcDNA6820431F20gene | | | | |  |
| 5730433K22Rik | 70532 | | 1,27 | | | 0,02 | | | RIKENcDNA5730433K22gene | | | | |  |
| Map3k12 | 26404 | | 1,27 | | | 0,05 | | | mitogenactivatedproteinkinasekinasekinase12 | | | | |  |
| Sdro | 70061 | | 1,27 | | | 0,03 | | | orphanshortchaindehydrogenase/reductase | | | | |  |
| Rag1 | 19373 | | 1,27 | | | 0,04 | | | recombinationactivatinggene1 | | | | |  |
| Cftr | 12638 | | 1,27 | | | 0,02 | | | cysticfibrosistransmembraneconductanceregulatorhomolog | | | | |  |
| 2010008C14Rik | 69859 | | 1,27 | | | 0,04 | | | RIKENcDNA2010008C14gene | | | | |  |
| Gpr109a | 80885 | | 1,27 | | | 0,02 | | | Gprotein-coupledreceptor109A | | | | |  |
| Hnrph2 | 56258 | | 1,27 | | | 0,03 | | | heterogeneousnuclearribonucleoproteinH2 | | | | |  |
| Aim1l | 230806 | | 1,27 | | | 0,03 | | | absentinmelanoma1-like | | | | |  |
| Sntb1 | 20649 | | 1,27 | | | 0,02 | | | syntrophin,basic1 | | | | |  |
| P2ry2 | 18442 | | 1,27 | | | 0,03 | | | purinergicreceptorP2Y,G-proteincoupled2 | | | | |  |
| Ddx3y | 26900 | | 1,27 | | | 0,01 | | | DEAD(Asp-Glu-Ala-Asp)boxpolypeptide3,Y-linked | | | | |  |
| Olfr801 | 258282 | | 1,26 | | | 0,02 | | | olfactoryreceptor801 | | | | |  |
| Stat6 | 20852 | | 1,26 | | | 0,04 | | | signaltransducerandactivatoroftranscription6 | | | | |  |
| Trpm1 | 17364 | | 1,26 | | | 0,03 | | | transientreceptorpotentialcationchannel,subfamilyM,member1 | | | | |  |
| Svs4 | 20941 | | 1,26 | | | 0,02 | | | seminalvesiclesecretoryprotein4 | | | | |  |
| 1500032F14Rik | 78305 | | 1,26 | | | 0,02 | | | RIKENcDNA1500032F14gene | | | | |  |
| 2310026E23Rik | 67621 | | 1,26 | | | 0,02 | | | RIKENcDNA2310026E23gene | | | | |  |
| Olfr374 | 258335 | | 1,25 | | | 0,02 | | | olfactoryreceptor374 | | | | |  |
| Krtap21-1 | 170656 | | 1,25 | | | 0,01 | | | keratinassociatedprotein16-7 | | | | |  |
| Zfp715 | 69930 | | 1,25 | | | 0,02 | | | zincfingerprotein715 | | | | |  |
| B930059L03Rik | 319786 | | 1,25 | | | 0,02 | | | RIKENcDNAB930059L03gene | | | | |  |
| Klra12 | 16630 | | 1,25 | | | 0,01 | | | killercelllectin-likereceptorsubfamilyA,member12 | | | | |  |
| D730048A03Rik | 77541 | | 1,25 | | | 0,03 | | | RIKENcDNAD730048A03gene | | | | |  |
| 1700010K24Rik | 75440 | | 1,25 | | | 0,01 | | | RIKENcDNA1700010K24gene | | | | |  |
| Spna1 | 20739 | | 1,25 | | | 0,02 | | | spectrinalpha1 | | | | |  |
| Matr3 | 17184 | | 1,25 | | | 0,01 | | | matrin3 | | | | |  |
| Olfr564 | 258356 | | 1,25 | | | 0,03 | | | olfactoryreceptor564 | | | | |  |
| Adi1 | 104923 | | 1,25 | | | 0,04 | | | acireductonedioxygenase1 | | | | |  |
| Cyp2c40 | 13099 | | 1,25 | | | 0,05 | | | cytochromeP450,family2,subfamilyc,polypeptide40 | | | | |  |
| Iigp1 | 60440 | | 1,25 | | | 0,01 | | | interferoninducibleGTPase1 | | | | |  |
| Zfp817 | 238693 | | 1,25 | | | 0,02 | | | zincfingerprotein817 | | | | |  |
| Trpm7 | 58800 | | 1,25 | | | 0,04 | | | transientreceptorpotentialcationchannel,subfamilyM,member7 | | | | |  |
| 9430083B18Rik | 77365 | | 1,25 | | | 0,02 | | | RIKENcDNA9430083B18gene | | | | |  |
| Asah2 | 54447 | | 1,25 | | | 0,02 | | | N-acylsphingosineamidohydrolase2 | | | | |  |
| 5033404E19Rik | 114668 | | 1,25 | | | 0,02 | | | RIKENcDNA5033404E19gene | | | | |  |
| Taok1 | 216965 | | 1,24 | | | 0,01 | | | TAOkinase1 | | | | |  |
| 4930547G20Rik | 75318 | | 1,24 | | | 0,02 | | | RIKENcDNA4930547G20gene | | | | |  |
| Chrnb4 | 108015 | | 1,24 | | | 0,04 | | | cholinergicreceptor,nicotinic,betapolypeptide4 | | | | |  |
| Tyms | 22171 | | 1,24 | | | 0,05 | | | thymidylatesynthase | | | | |  |
| Pabpc1 | 18458 | | 1,24 | | | 0,04 | | | polyAbindingprotein,cytoplasmic1 | | | | |  |
| 4930428N03Rik | 73871 | | 1,24 | | | 0,01 | | | RIKENcDNA4930428N03gene | | | | |  |
| Ttc1 | 66827 | | 1,24 | | | 0,01 | | | tetratricopeptiderepeatdomain1 | | | | |  |
| Cys-ARNt_TRNC | 4511 | | 1,24 | | | 0,00 | | | mitochondriallyencodedtRNAcysteine[Homosapiens] | | | | |  |
| Adamts20 | 223838 | | 1,24 | | | 0,01 | | | adisintegrin-likeandmetallopeptidase(reprolysintype)withthrombospondintype1motif,20 | | | | |  |
| Olfr1132 | 258833 | | 1,24 | | | 0,01 | | | olfactoryreceptor1132 | | | | |  |
| Prl2b1 | 66392 | | 1,24 | | | 0,02 | | | prolactinfamily2,subfamilyb,member1 | | | | |  |
| Itgb1 | 16412 | | 1,24 | | | 0,05 | | | integrinbeta1(fibronectinreceptorbeta) | | | | |  |
| Rnf11 | 29864 | | 1,24 | | | 0,03 | | | ringfingerprotein11 | | | | |  |
| Mrcl | 353287 | | 1,24 | | | 0,01 | | | mannosereceptor-likeprecursor | | | | |  |
| Olfr1176 | 258767 | | 1,24 | | | 0,01 | | | olfactoryreceptor1176 | | | | |  |
| Pus3 | 67049 | | 1,24 | | | 0,05 | | | pseudouridinesynthase3 | | | | |  |
| Cage1 | 71213 | | 1,24 | | | 0,05 | | | cancerantigen1 | | | | |  |
| Morf4l2 | 56397 | | 1,24 | | | 0,01 | | | mortalityfactor4like2 | | | | |  |
| Crct1 | 74175 | | 1,23 | | | 0,02 | | | cysteine-richC-terminal1 | | | | |  |
| Zyg11a | 230590 | | 1,23 | | | 0,00 | | | zyg-11homologA(C.elegans) | | | | |  |
| A530050D06Rik | 104816 | | 1,23 | | | 0,02 | | | RIKENcDNAA530050D06gene | | | | |  |
| E330034G19Rik | 105418 | | 1,23 | | | 0,00 | | | RIKENcDNAE330034G19gene | | | | |  |
| B2m | 12010 | | 1,23 | | | 0,00 | | | beta-2microglobulin | | | | |  |
| Usp28 | 235323 | | 1,23 | | | 0,02 | | | ubiquitinspecificpeptidase28 | | | | |  |
| Mtpn | 14489 | | 1,23 | | | 0,01 | | | myotrophin | | | | |  |
| Aqr | 11834 | | 1,23 | | | 0,02 | | | aquarius | | | | |  |
| Slc46a1 | 52466 | | 1,23 | | | 0,03 | | | solutecarrierfamily46,member1 | | | | |  |
| A930036I15Rik | 77967 | | 1,23 | | | 0,01 | | | RIKENcDNAA930036I15gene | | | | |  |
| Rtl1 | 353326 | | 1,23 | | | 0,02 | | | retrotransposon-like1 | | | | |  |
| Olfr1160 | 258643 | | 1,22 | | | 0,00 | | | olfactoryreceptor1160 | | | | |  |
| Bapx1 | 12020 | | 1,22 | | | 0,00 | | | NK3homeobox2 | | | | |  |
| Bhlhb5 | 59058 | | 1,22 | | | 0,03 | | | basichelix-loop-helixdomaincontaining,classB5 | | | | |  |
| 1700021A07Rik | 76924 | | 1,22 | | | 0,02 | | | RIKENcDNA1700021A07gene | | | | |  |
| Mdh1 | 17449 | | 1,22 | | | 0,01 | | | malatedehydrogenase1,NAD(soluble) | | | | |  |
| Gtpbp6 | 107999 | | 1,22 | | | 0,02 | | | GTPbindingprotein6(putative) | | | | |  |
| 5730403B10Rik | 66626 | | 1,22 | | | 0,00 | | | RIKENcDNA5730403B10gene | | | | |  |
| B230120H23Rik | 65964 | | 1,22 | | | 0,01 | | | RIKENcDNAB230120H23gene | | | | |  |
| Fabp5 | 16592 | | 1,22 | | | 0,01 | | | fattyacidbindingprotein5,epidermal | | | | |  |
| 9530077C05Rik | 68283 | | 1,22 | | | 0,01 | | | RIKENcDNA9530077C05gene | | | | |  |
| Sumo2 | 170930 | | 1,22 | | | 0,02 | | | SMT3suppressorofmiftwo3homolog2(yeast) | | | | |  |
| Golph4 | 73124 | | 1,22 | | | 0,05 | | | golgiintegralmembraneprotein4 | | | | |  |
| Wasf1 | 83767 | | 1,22 | | | 0,04 | | | WASPfamily1 | | | | |  |
| Rgl1 | 19731 | | 1,22 | | | 0,02 | | | ralguaninenucleotidedissociationstimulator,-like1 | | | | |  |
| C1qb | 12260 | | 1,22 | | | 0,05 | | | complementcomponent1,qsubcomponent,betapolypeptide | | | | |  |
| Asb7 | 117589 | | 1,22 | | | 0,01 | | | ankyrinrepeatandSOCSbox-containingprotein7 | | | | |  |
| Olfr572 | 259093 | | 1,22 | | | 0,04 | | | olfactoryreceptor572 | | | | |  |
| Pcnx | 54604 | | 1,22 | | | 0,02 | | | pecanexhomolog(Drosophila) | | | | |  |
| C130057D23Rik | 328505 | | 1,22 | | | 0,01 | | | RIKENcDNAC130057D23gene | | | | |  |
| Ift80 | 68259 | | 1,22 | | | 0,03 | | | intraflagellartransport80homolog(Chlamydomonas) | | | | |  |
| Wnt7a | 22421 | | 1,21 | | | 0,02 | | | wingless-relatedMMTVintegrationsite7A | | | | |  |
| Ikzf3 | 22780 | | 1,21 | | | 0,04 | | | IKAROSfamilyzincfinger3 | | | | |  |
| Ptp4a1 | 19243 | | 1,21 | | | 0,05 | | | proteintyrosinephosphatase4a1 | | | | |  |
| 4930449I24Rik | 67410 | | 1,21 | | | 0,03 | | | RIKENcDNA4930449I24gene | | | | |  |
| Rgs7 | 24012 | | 1,21 | | | 0,01 | | | regulatorofGproteinsignaling7 | | | | |  |
| C77370 | 245555 | | 1,21 | | | 0,00 | | | expressedsequenceC77370 | | | | |  |
| Rpl39 | 67248 | | 1,21 | | | 0,04 | | | ribosomalproteinL39 | | | | |  |
| Ptchd1 | 211612 | | 1,21 | | | 0,00 | | | patcheddomaincontaining1 | | | | |  |
| V1re7 | 171230 | | 1,21 | | | 0,02 | | | vomeronasal1receptor,E7 | | | | |  |
| Camk2b | 12323 | | 1,21 | | | 0,05 | | | calcium/calmodulin-dependentproteinkinaseII,beta | | | | |  |
| Slc11a2 | 18174 | | 1,21 | | | 0,01 | | | solutecarrierfamily11(proton-coupleddivalentmetaliontransporters),member2 | | | | |  |
| Asz1 | 74068 | | 1,21 | | | 0,00 | | | ankyrinrepeat,SAMandbasicleucinezipperdomaincontaining1 | | | | |  |
| 2310016C16Rik | 69590 | | 1,21 | | | 0,05 | | | RIKENcDNA2310016C16gene | | | | |  |
| Hsp90b1 | 22027 | | 1,21 | | | 0,01 | | | heatshockprotein90kDabeta(Grp94),member1 | | | | |  |
| Actr3b | 242894 | | 1,21 | | | 0,02 | | | ARP3actin-relatedprotein3homologB(yeast) | | | | |  |
| Shmt2 | 108037 | | 1,21 | | | 0,02 | | | serinehydroxymethyltransferase2(mitochondrial) | | | | |  |
| AY074887 | 246735 | | 1,21 | | | 0,02 | | | cDNAsequenceAY074887 | | | | |  |
| 1700010B13Rik | 75491 | | 1,21 | | | 0,01 | | | RIKENcDNA1700010B13gene | | | | |  |
| 1700016C23Rik | 76931 | | 1,21 | | | 0,05 | | | RIKENcDNA1700016C23gene | | | | |  |
| H2-D1 | 14964 | | 1,21 | | | 0,01 | | | histocompatibility2,Dregionlocus1 | | | | |  |
| Pak6 | 214230 | | 1,21 | | | 0,01 | | | p21(CDKN1A)-activatedkinase6 | | | | |  |
| Rnf208 | 68846 | | 1,21 | | | 0,02 | | | ringfingerprotein208 | | | | |  |
| Kcnk13 | 217826 | | 1,21 | | | 0,01 | | | potassiumchannel,subfamilyK,member13 | | | | |  |
| Dmrt1 | 50796 | | 1,20 | | | 0,01 | | | doublesexandmab-3relatedtranscriptionfactor1 | | | | |  |
| Il33 | 77125 | | 1,20 | | | 0,01 | | | interleukin33 | | | | |  |
| EG235327 | 235327 | | 1,20 | | | 0,00 | | | predictedgene,EG235327 | | | | |  |
| Phyhipl | 70911 | | 1,20 | | | 0,03 | | | phytanoyl-CoAhydroxylaseinteractingprotein-like | | | | |  |
| CYP19A1 | 1588 | | 1,20 | | | 0,04 | | | cytochromeP450,family19,subfamilyA,polypeptide1[Homosapiens] | | | | |  |
| Sbno1 | 243272 | | 1,20 | | | 0,02 | | | sno,strawberrynotchhomolog1(Drosophila) | | | | |  |
| Acbd3 | 170760 | | 1,20 | | | 0,01 | | | acyl-CoenzymeAbindingdomaincontaining3 | | | | |  |
| Rpl26 | 19941 | | 1,20 | | | 0,03 | | | ribosomalproteinL26 | | | | |  |
| Thap7 | 69009 | | 1,20 | | | 0,00 | | | THAPdomaincontaining7 | | | | |  |
| Nin | 18080 | | 1,20 | | | 0,04 | | | ninein | | | | |  |
| Rps15a | 267019 | | 1,20 | | | 0,02 | | | ribosomalproteinS15a | | | | |  |
| Nphp3 | 74025 | | 1,20 | | | 0,01 | | | nephronophthisis3(adolescent) | | | | |  |
| Tegt | 110213 | | 1,20 | | | 0,02 | | | testisenhancedgenetranscript | | | | |  |
| Gpr18 | 110168 | | 1,20 | | | 0,03 | | | Gprotein-coupledreceptor18 | | | | |  |
| Dhrs7 | 66375 | | 1,20 | | | 0,00 | | | dehydrogenase/reductase(SDRfamily)member7 | | | | |  |
| Tmem169 | 271711 | | 1,20 | | | 0,03 | | | transmembraneprotein169 | | | | |  |
| Wt1 | 22431 | | 1,20 | | | 0,00 | | | Wilmstumorhomolog | | | | |  |
| Foxd1 | 15229 | | 1,20 | | | 0,02 | | | forkheadboxD1 | | | | |  |
| BC031748 | 245622 | | 1,20 | | | 0,01 | | | cDNAsequenceBC031748 | | | | |  |
| Fgfbp3 | 72514 | | 1,19 | | | 0,02 | | | fibroblastgrowthfactorbindingprotein3 | | | | |  |
| U2af1l4 | 233073 | | 1,19 | | | 0,03 | | | U2smallnuclearRNAauxiliaryfactor1-like4 | | | | |  |
| Gpr126 | 215798 | | 1,19 | | | 0,01 | | | Gprotein-coupledreceptor126 | | | | |  |
| Myh4 | 17884 | | 1,19 | | | 0,03 | | | myosin,heavypolypeptide4,skeletalmuscle | | | | |  |
| E130102C15Rik | 77995 | | 1,19 | | | 0,03 | | | RIKENcDNAE130102C15gene | | | | |  |
| 1700034H14Rik | 67105 | | 1,19 | | | 0,04 | | | RIKENcDNA1700034H14gene | | | | |  |
| Olfr1423 | 258675 | | 1,19 | | | 0,04 | | | olfactoryreceptor1423 | | | | |  |
| Prdm5 | 70779 | | 1,19 | | | 0,03 | | | PRdomaincontaining5 | | | | |  |
| Phtf1 | 18685 | | 1,19 | | | 0,03 | | | putativehomeodomaintranscriptionfactor1 | | | | |  |
| Dep1 | 170624 | | 1,19 | | | 0,02 | | | diabeticembryopathy1 | | | | |  |
| Gpr75 | 237716 | | 1,19 | | | 0,00 | | | Gprotein-coupledreceptor75 | | | | |  |
| Hrh1 | 15465 | | 1,19 | | | 0,03 | | | histaminereceptorH1 | | | | |  |
| Map2k1ip1 | 56692 | | 1,19 | | | 0,01 | | | mitogen-activatedproteinkinasekinase1interactingprotein1 | | | | |  |
| Dhcr7 | 13360 | | 1,19 | | | 0,05 | | | 7-dehydrocholesterolreductase | | | | |  |
| Ppp1cb | 19046 | | 1,19 | | | 0,04 | | | proteinphosphatase1,catalyticsubunit,betaisoform | | | | |  |
| 9530003J23Rik | 77397 | | 1,19 | | | 0,02 | | | RIKENcDNA9530003J23gene | | | | |  |
| Akna | 100182 | | 1,18 | | | 0,04 | | | AT-hooktranscriptionfactor | | | | |  |
| 1700011F14Rik | 75645 | | 1,18 | | | 0,04 | | | RIKENcDNA1700011F14gene | | | | |  |
| COX1 | 4512 | | 1,18 | | | 0,01 | | | mitochondriallyencodedcytochromecoxidaseI[Homosapiens] | | | | |  |
| Iqgap1 | 29875 | | 1,18 | | | 0,01 | | | IQmotifcontainingGTPaseactivatingprotein1 | | | | |  |
| BC003965 | 214489 | | 1,18 | | | 0,03 | | | cDNAsequenceBC003965 | | | | |  |
| 9330168M11Rik | 320839 | | 1,18 | | | 0,03 | | | RIKENcDNA9330168M11gene | | | | |  |
| 0610025J13Rik | 78687 | | 1,18 | | | 0,02 | | | RIKENcDNA0610025J13gene | | | | |  |
| Taar1 | 111174 | | 1,18 | | | 0,03 | | | traceamine-associatedreceptor1 | | | | |  |
| Mbd3l2 | 234988 | | 1,18 | | | 0,02 | | | methyl-CpGbindingdomainprotein3-like2 | | | | |  |
| 2600005O03Rik | 72107 | | 1,18 | | | 0,01 | | | RIKENcDNA2600005O03gene | | | | |  |
| Fancd2 | 211651 | | 1,18 | | | 0,00 | | | Fanconianemia,complementationgroupD2 | | | | |  |
| Ankrd50 | 99696 | | 1,18 | | | 0,02 | | | ankrinrepeatdomain50 | | | | |  |
| Baiap2l2 | 207495 | | 1,18 | | | 0,01 | | | BAI1-associatedprotein2-like2 | | | | |  |
| March2 | 224703 | | 1,18 | | | 0,04 | | | membrane-associatedringfinger(C3HC4)2 | | | | |  |
| Olfr887 | 258415 | | 1,18 | | | 0,04 | | | olfactoryreceptor887 | | | | |  |
| Kbtbd7 | 211255 | | 1,18 | | | 0,02 | | | kelchrepeatandBTB(POZ)domaincontaining7 | | | | |  |
| Hmgcl | 15356 | | 1,18 | | | 0,03 | | | 3-hydroxy-3-methylglutaryl-CoenzymeAlyase | | | | |  |
| Krt17 | 16667 | | 1,18 | | | 0,04 | | | keratin17 | | | | |  |
| Dip2c | 208440 | | 1,18 | | | 0,03 | | | DIP2disco-interactingprotein2homologC(Drosophila) | | | | |  |
| Nptn | 20320 | | 1,18 | | | 0,03 | | | neuroplastin | | | | |  |
| 6330444E15Rik | 442797 | | 1,18 | | | 0,05 | | | RIKENcDNA6330444E15gene | | | | |  |
| Acot9 | 56360 | | 1,18 | | | 0,05 | | | acyl-CoAthioesterase9 | | | | |  |
| 1810037I17Rik | 67704 | | 1,18 | | | 0,01 | | | RIKENcDNA1810037I17gene | | | | |  |
| Frat2 | 212398 | | 1,18 | | | 0,04 | | | frequentlyrearrangedinadvancedT-celllymphomas2 | | | | |  |
| 4930567H12Rik | 75930 | | 1,18 | | | 0,02 | | | RIKENcDNA4930567H12gene | | | | |  |
| Irf6 | 54139 | | 1,18 | | | 0,04 | | | interferonregulatoryfactor6 | | | | |  |
| Ppia | 268373 | | 1,18 | | | 0,02 | | | peptidylprolylisomeraseA | | | | |  |
| Fstl1 | 14314 | | 1,18 | | | 0,03 | | | follistatin-like1 | | | | |  |
| Efnb3 | 13643 | | 1,18 | | | 0,05 | | | ephrinB3 | | | | |  |
| 3110035C09Rik | 73161 | | 1,18 | | | 0,01 | | | RIKENcDNA3110035C09gene | | | | |  |
| Hist1h2ba | 319177 | | 1,18 | | | 0,02 | | | histonecluster1,H2ba | | | | |  |
| Prune | 229589 | | 1,18 | | | 0,04 | | | prunehomolog(Drosophila) | | | | |  |
| A230062G08Rik | 231326 | | 1,18 | | | 0,04 | | | RIKENcDNAA230062G08gene | | | | |  |
| 1700030J22Rik | 69528 | | 1,17 | | | 0,03 | | | RIKENcDNA1700030J22gene | | | | |  |
| Tmsb4x | 19241 | | 1,17 | | | 0,00 | | | thymosin,beta4,Xchromosome | | | | |  |
| Top1 | 21969 | | 1,17 | | | 0,01 | | | topoisomerase(DNA)I | | | | |  |
| Dusp11 | 72102 | | 1,17 | | | 0,03 | | | dualspecificityphosphatase11(RNA/RNPcomplex1-interacting) | | | | |  |
| Olfr933 | 258433 | | 1,17 | | | 0,00 | | | olfactoryreceptor933 | | | | |  |
| Sucla2 | 20916 | | 1,17 | | | 0,01 | | | succinate-CoenzymeAligase,ADP-forming,betasubunit | | | | |  |
| Dpp3 | 75221 | | 1,17 | | | 0,05 | | | dipeptidylpeptidase3 | | | | |  |
| 1500031L02Rik | 66994 | | 1,17 | | | 0,01 | | | RIKENcDNA1500031L02gene | | | | |  |
| Dnalc1 | 105000 | | 1,17 | | | 0,03 | | | dynein,axonemal,lightchain1 | | | | |  |
| Egfl6 | 54156 | | 1,17 | | | 0,04 | | | EGF-like-domain,multiple6 | | | | |  |
| 1110013L07Rik | 68521 | | 1,17 | | | 0,00 | | | RIKENcDNA1110013L07gene | | | | |  |
| Dcx | 13193 | | 1,17 | | | 0,01 | | | doublecortin | | | | |  |
| Fbxw2 | 30050 | | 1,17 | | | 0,04 | | | F-boxandWD-40domainprotein2 | | | | |  |
| Rdh14 | 105014 | | 1,17 | | | 0,01 | | | retinoldehydrogenase14(all-transand9-cis) | | | | |  |
| Pap | 18489 | | 1,17 | | | 0,01 | | | pancreatitis-associatedprotein | | | | |  |
| Creb3l1 | 26427 | | 1,17 | | | 0,04 | | | cAMPresponsiveelementbindingprotein3-like1 | | | | |  |
| Actr1a | 54130 | | 1,17 | | | 0,04 | | | ARP1actin-relatedprotein1homologA(yeast) | | | | |  |
| Cand1 | 71902 | | 1,17 | | | 0,03 | | | cullinassociatedandneddylationdisassociated1 | | | | |  |
| Clic4 | 29876 | | 1,17 | | | 0,02 | | | chlorideintracellularchannel4(mitochondrial) | | | | |  |
| Prlr | 19116 | | 1,17 | | | 0,03 | | | prolactinreceptor | | | | |  |
| Zfp558 | 72230 | | 1,17 | | | 0,03 | | | zincfingerprotein558 | | | | |  |
| Trip12 | 14897 | | 1,17 | | | 0,00 | | | thyroidhormonereceptorinteractor12 | | | | |  |
| Spats1 | 71020 | | 1,17 | | | 0,01 | | | spermatogenesisassociated,serine-rich1 | | | | |  |
| Rnf122 | 68867 | | 1,16 | | | 0,04 | | | ringfingerprotein122 | | | | |  |
| 8430436O14Rik | 71506 | | 1,16 | | | 0,04 | | | RIKENcDNA8430436O14gene | | | | |  |
| Herc1 | 235439 | | 1,16 | | | 0,05 | | | hect(homologoustotheE6-AP(UBE3A)carboxylterminus)domainandRCC1(CHC1)-likedomain(RLD)1 | | | | |  |
| Serpinh1 | 12406 | | 1,16 | | | 0,05 | | | serine(orcysteine)peptidaseinhibitor,cladeH,member1 | | | | |  |
| Hnrpl | 15388 | | 1,16 | | | 0,03 | | | heterogeneousnuclearribonucleoproteinL | | | | |  |
| Atad2b | 320817 | | 1,16 | | | 0,01 | | | ATPasefamily,AAAdomaincontaining2B | | | | |  |
| 4933417O08Rik | 71146 | | 1,16 | | | 0,03 | | | RIKENcDNA4933417O08gene | | | | |  |
| Rwdd1 | 66521 | | 1,16 | | | 0,03 | | | RWDdomaincontaining1 | | | | |  |
| 1810010M01Rik | 69036 | | 1,16 | | | 0,02 | | | RIKENcDNA1810010M01gene | | | | |  |
| Top1mt | 72960 | | 1,16 | | | 0,03 | | | DNAtopoisomerase1,mitochondrial | | | | |  |
| 4930431P22Rik | 73883 | | 1,16 | | | 0,03 | | | RIKENcDNA4930431P22gene | | | | |  |
| 2700089E24Rik | 381820 | | 1,16 | | | 0,01 | | | RIKENcDNA2700089E24gene | | | | |  |
| Plcxd3 | 239318 | | 1,16 | | | 0,00 | | | phosphatidylinositol-specificphospholipaseC,Xdomaincontaining3 | | | | |  |
| Lao1 | 100470 | | 1,16 | | | 0,01 | | | L-aminoacidoxidase1 | | | | |  |
| Fh1 | 14194 | | 1,16 | | | 0,02 | | | fumaratehydratase1 | | | | |  |
| Gpr173 | 70771 | | 1,16 | | | 0,05 | | | G-proteincoupledreceptor173 | | | | |  |
| Dio1 | 13370 | | 1,16 | | | 0,01 | | | deiodinase,iodothyronine,typeI | | | | |  |
| Lhb | 16866 | | 1,16 | | | 0,04 | | | luteinizinghormonebeta | | | | |  |
| Atp8a1 | 11980 | | 1,16 | | | 0,04 | | | ATPase,aminophospholipidtransporter(APLT),classI,type8A,member1 | | | | |  |
| Prkar1a | 19084 | | 1,16 | | | 0,01 | | | proteinkinase,cAMPdependentregulatory,typeI,alpha | | | | |  |
| Mett5d1 | 76894 | | 1,16 | | | 0,03 | | | methyltransferase5domaincontaining1 | | | | |  |
| Esrrb | 26380 | | 1,16 | | | 0,02 | | | estrogenrelatedreceptor,beta | | | | |  |
| Kctd16 | 383348 | | 1,16 | | | 0,03 | | | potassiumchanneltetramerisationdomaincontaining16 | | | | |  |
| A930005I04Rik | 403174 | | 1,16 | | | 0,05 | | | RIKENcDNAA930005I04gene | | | | |  |
| 0610037P05Rik | 66086 | | 1,16 | | | 0,05 | | | RIKENcDNA0610037P05gene | | | | |  |
| Olfr656 | 259078 | | 1,16 | | | 0,03 | | | olfactoryreceptor656 | | | | |  |
| Tram1l1 | 229801 | | 1,16 | | | 0,04 | | | translocationassociatedmembraneprotein1-like1 | | | | |  |
| 4933425M15Rik | 319217 | | 1,16 | | | 0,05 | | | RIKENcDNA4933425M15gene | | | | |  |
| 4930447I22Rik | 112413 | | 1,15 | | | 0,01 | | | RIKENcDNA4930447I22gene | | | | |  |
| Mettl7b | 71664 | | 1,15 | | | 0,03 | | | methyltransferaselike7B | | | | |  |
| Myh3 | 17883 | | 1,15 | | | 0,04 | | | myosin,heavypolypeptide3,skeletalmuscle,embryonic | | | | |  |
| Pgk2 | 18663 | | 1,15 | | | 0,01 | | | phosphoglyceratekinase2 | | | | |  |
| Eif2b2 | 217715 | | 1,15 | | | 0,03 | | | eukaryotictranslationinitiationfactor2B,subunit2beta | | | | |  |
| Bivm | 246229 | | 1,15 | | | 0,05 | | | basic,immunoglobulin-likevariablemotifcontaining | | | | |  |
| Nfam1 | 74039 | | 1,15 | | | 0,01 | | | NfatactivatingmoleculewithITAMmotif1 | | | | |  |
| Zfp28 | 22690 | | 1,15 | | | 0,01 | | | zincfingerprotein28 | | | | |  |
| Olfr178 | 258999 | | 1,15 | | | 0,02 | | | olfactoryreceptor178 | | | | |  |
| Ttc28 | 209683 | | 1,15 | | | 0,02 | | | tetratricopeptiderepeatdomain28 | | | | |  |
| Dnaic1 | 68922 | | 1,15 | | | 0,02 | | | dynein,axonemal,intermediatechain1 | | | | |  |
| Olfr273 | 258821 | | 1,15 | | | 0,02 | | | olfactoryreceptor273 | | | | |  |
| 5830420C07Rik | 77599 | | 1,15 | | | 0,03 | | | RIKENcDNA5830420C07gene | | | | |  |
| Acvr1 | 11477 | | 1,15 | | | 0,03 | | | activinAreceptor,type1 | | | | |  |
| COX3 | 4514 | | 1,15 | | | 0,03 | | | mitochondriallyencodedcytochromecoxidaseIII[Homosapiens] | | | | |  |
| Zfp462 | 242466 | | 1,15 | | | 0,02 | | | zincfingerprotein462 | | | | |  |
| Eif1ay | 66235 | | 1,15 | | | 0,01 | | | eukaryotictranslationinitiationfactor1A,Y-linked | | | | |  |
| 8430431K14Rik | 78103 | | 1,15 | | | 0,03 | | | RIKENcDNA8430431K14gene | | | | |  |
| Ddx41 | 72935 | | 1,15 | | | 0,03 | | | DEAD(Asp-Glu-Ala-Asp)boxpolypeptide41 | | | | |  |
| Abca15 | 320631 | | 1,15 | | | 0,04 | | | ATP-bindingcassette,sub-familyA(ABC1),member15 | | | | |  |
| Sh3yl1 | 24057 | | 1,15 | | | 0,04 | | | Sh3domainYSC-like1 | | | | |  |
| Rif1 | 51869 | | 1,15 | | | 0,03 | | | Rap1interactingfactor1homolog(yeast) | | | | |  |
| Rbbp9 | 26450 | | 1,15 | | | 0,03 | | | retinoblastomabindingprotein9 | | | | |  |
| Cd2 | 12481 | | 1,15 | | | 0,04 | | | CD2antigen | | | | |  |
| Junb | 16477 | | 1,15 | | | 0,01 | | | Jun-Boncogene | | | | |  |
| EG269902 | 269902 | | 1,15 | | | 0,04 | | | predictedgene,EG269902 | | | | |  |
| Adamts8 | 30806 | | 1,15 | | | 0,02 | | | adisintegrin-likeandmetallopeptidase(reprolysintype)withthrombospondintype1motif,8 | | | | |  |
| Cdc26 | 66440 | | 1,15 | | | 0,01 | | | celldivisioncycle26 | | | | |  |
| Utx | 22289 | | 1,15 | | | 0,03 | | | ubiquitouslytranscribedtetratricopeptiderepeatgene,Xchromosome | | | | |  |
| Mgst1 | 56615 | | 1,15 | | | 0,01 | | | microsomalglutathioneS-transferase1 | | | | |  |
| 1700016H03Rik | 75653 | | 1,15 | | | 0,03 | | | RIKENcDNA1700016H03gene | | | | |  |
| 1700010K23Rik | 75501 | | 1,15 | | | 0,01 | | | RIKENcDNA1700010K23gene | | | | |  |
| Gnb4 | 14696 | | 1,15 | | | 0,05 | | | guaninenucleotidebindingprotein,beta4 | | | | |  |
| BC055004 | 381680 | | 1,15 | | | 0,03 | | | cDNAsequenceBC055004 | | | | |  |
| 5830411K21Rik | 78822 | | 1,15 | | | 0,04 | | | RIKENcDNA5830411K21gene | | | | |  |
| Pxn | 19303 | | 1,15 | | | 0,05 | | | paxillin | | | | |  |
| Igk-V28 | 16114 | | 1,15 | | | 0,01 | | | immunoglobulinkappachainvariable28(V28) | | | | |  |
| Tipin | 66131 | | 1,15 | | | 0,01 | | | timelessinteractingprotein | | | | |  |
| Ccdc121 | 403180 | | 1,15 | | | 0,02 | | | coiled-coildomaincontaining121 | | | | |  |
| Pnpla8 | 67452 | | 1,15 | | | 0,02 | | | patatin-likephospholipasedomaincontaining8 | | | | |  |
| Mitd1 | 69028 | | 1,15 | | | 0,01 | | | MIT,microtubuleinteractingandtransport,domaincontaining1 | | | | |  |
| A030009A09Rik | 77763 | | 1,15 | | | 0,03 | | | RIKENcDNAA030009A09gene | | | | |  |
| Eif4g2 | 13690 | | 1,15 | | | 0,01 | | | eukaryotictranslationinitiationfactor4,gamma2 | | | | |  |
| Ppap2c | 50784 | | 1,15 | | | 0,03 | | | phosphatidicacidphosphatasetype2c | | | | |  |
| Pgm5 | 226041 | | 1,15 | | | 0,00 | | | phosphoglucomutase5 | | | | |  |
| 4933415J04Rik | 71172 | | 1,15 | | | 0,04 | | | RIKENcDNA4933415J04gene | | | | |  |
| Tnfrsf18 | 21936 | | 1,15 | | | 0,05 | | | tumornecrosisfactorreceptorsuperfamily,member18 | | | | |  |
| Stfa2l1 | 268885 | | 1,15 | | | 0,04 | | | stefinA2like1 | | | | |  |
| Aktip | 14339 | | 1,14 | | | 0,00 | | | thymomaviralproto-oncogene1interactingprotein | | | | |  |
| Pias3 | 229615 | | 1,14 | | | 0,00 | | | proteininhibitorofactivatedSTAT3 | | | | |  |
| Lgmn | 19141 | | 1,14 | | | 0,05 | | | legumain | | | | |  |
| Col4a3bp | 68018 | | 1,14 | | | 0,01 | | | procollagen,typeIV,alpha3(Goodpastureantigen)bindingprotein | | | | |  |
| EG245190 | 245190 | | 1,14 | | | 0,02 | | | predictedgene,EG245190 | | | | |  |
| 5830435N06Rik | 76033 | | 1,14 | | | 0,01 | | | RIKENcDNA5830435N06gene | | | | |  |
| 4930595M18Rik | 245492 | | 1,14 | | | 0,03 | | | RIKENcDNA4930595M18gene | | | | |  |
| S100a4 | 20198 | | 1,14 | | | 0,01 | | | S100calciumbindingproteinA4 | | | | |  |
| Tex264 | 21767 | | 1,14 | | | 0,03 | | | testisexpressedgene264 | | | | |  |
| Ndufc2 | 68197 | | 1,14 | | | 0,04 | | | NADHdehydrogenase(ubiquinone)1,subcomplexunknown,2 | | | | |  |
| Olfr1447 | 258698 | | 1,14 | | | 0,03 | | | olfactoryreceptor1447 | | | | |  |
| Dph4 | 99349 | | 1,14 | | | 0,01 | | | DPH4homolog(JJJ3,S.cerevisiae) | | | | |  |
| 6530437J22Rik | 286968 | | 1,14 | | | 0,02 | | | RIKENcDNA6530437J22gene | | | | |  |
| Mrps18b | 66973 | | 1,14 | | | 0,00 | | | mitochondrialribosomalproteinS18B | | | | |  |
| Klhl1 | 93688 | | 1,14 | | | 0,03 | | | kelch-like1(Drosophila) | | | | |  |
| 4930563I02Rik | 67659 | | 1,14 | | | 0,02 | | | RIKENcDNA4930563I02gene | | | | |  |
| Ndufs1 | 227197 | | 1,14 | | | 0,00 | | | NADHdehydrogenase(ubiquinone)Fe-Sprotein1 | | | | |  |
| Fgf11 | 14166 | | 1,14 | | | 0,05 | | | fibroblastgrowthfactor11 | | | | |  |
| Dock5 | 68813 | | 1,14 | | | 0,01 | | | dedicatorofcytokinesis5 | | | | |  |
| Trim59 | 66949 | | 1,14 | | | 0,03 | | | tripartitemotif-containing59 | | | | |  |
| 4933438K21Rik | 71270 | | 1,14 | | | 0,04 | | | RIKENcDNA4933438K21gene | | | | |  |
| Usp1 | 230484 | | 1,14 | | | 0,01 | | | ubiquitinspecificpeptdiase1 | | | | |  |
| Amotl2 | 56332 | | 1,14 | | | 0,01 | | | angiomotinlike2 | | | | |  |
| H2-Oa | 15001 | | 1,14 | | | 0,03 | | | histocompatibility2,Oregionalphalocus | | | | |  |
| Igk-V1 | 16081 | | 1,14 | | | 0,02 | | | immunoglobulinkappachainvariable1(V1) | | | | |  |
| Rreb1 | 68750 | | 1,14 | | | 0,02 | | | rasresponsiveelementbindingprotein1 | | | | |  |
| Hsp90aa1 | 15519 | | 1,14 | | | 0,01 | | | heatshockprotein90kDaalpha(cytosolic),classAmember1 | | | | |  |
| Spcs3 | 76687 | | 1,14 | | | 0,04 | | | signalpeptidasecomplexsubunit3homolog(S.cerevisiae) | | | | |  |
| 4930401O12Rik | 73924 | | 1,14 | | | 0,01 | | | RIKENcDNA4930401O12gene | | | | |  |
| 6430597G12Rik | 76210 | | 1,14 | | | 0,04 | | | RIKENcDNA6430597G12gene | | | | |  |
| Skil | 20482 | | 1,14 | | | 0,01 | | | SKI-like | | | | |  |
| Rtn4 | 68585 | | 1,14 | | | 0,03 | | | reticulon4 | | | | |  |
| Trp63 | 22061 | | 1,14 | | | 0,03 | | | transformationrelatedprotein63 | | | | |  |
| Vpreb3 | 22364 | | 1,14 | | | 0,04 | | | pre-Blymphocytegene3 | | | | |  |
| Pkn3 | 263803 | | 1,14 | | | 0,00 | | | proteinkinaseN3 | | | | |  |
| Khdrbs1 | 20218 | | 1,14 | | | 0,03 | | | KHdomaincontaining,RNAbinding,signaltransductionassociated1 | | | | |  |
| PROK2 | 60675 | | 1,14 | | | 0,00 | | | prokineticin2[Homosapiens] | | | | |  |
| C330021F23Rik | 546049 | | 1,13 | | | 0,04 | | | RIKENcDNAC330021F23gene | | | | |  |
| Olfr186 | 258318 | | 1,13 | | | 0,00 | | | olfactoryreceptor186 | | | | |  |
| Pkd2l1 | 329064 | | 1,13 | | | 0,02 | | | polycystickidneydisease2-like1 | | | | |  |
| Galk2 | 69976 | | 1,13 | | | 0,03 | | | galactokinase2 | | | | |  |
| Dock11 | 75974 | | 1,13 | | | 0,03 | | | dedicatorofcytokinesis11 | | | | |  |
| 4930569F06Rik | 78055 | | 1,13 | | | 0,01 | | | RIKENcDNA4930569F06gene | | | | |  |
| 5830457O10Rik | 214987 | | 1,13 | | | 0,04 | | | RIKENcDNA5830457O10gene | | | | |  |
| Spink5 | 72432 | | 1,13 | | | 0,04 | | | serinepeptidaseinhibitor,Kazaltype5 | | | | |  |
| Ccdc59 | 52713 | | 1,13 | | | 0,00 | | | coiled-coildomaincontaining59 | | | | |  |
| Mylk2 | 228785 | | 1,13 | | | 0,02 | | | myosin,lightpolypeptidekinase2,skeletalmuscle | | | | |  |
| 3110037B15Rik | 73164 | | 1,13 | | | 0,05 | | | RIKENcDNA3110037B15gene | | | | |  |
| 6430706D22Rik | 381280 | | 1,13 | | | 0,04 | | | RIKENcDNA6430706D22gene | | | | |  |
| Cyp27a1 | 104086 | | 1,13 | | | 0,00 | | | cytochromeP450,family27,subfamilya,p | | | | |  |
